# Cost Effectiveness of Free Access to Smoking Cessation Treatment in France Considering the Economic Burden of Smoking-Related Diseases

**DOI:** 10.1371/journal.pone.0148750

**Published:** 2016-02-24

**Authors:** Benjamin Cadier, Isabelle Durand-Zaleski, Daniel Thomas, Karine Chevreul

**Affiliations:** 1 AP-HP URC-Eco Ile-de-France, Paris, France; 2 Inserm, ECEVE, U1123, Paris, France; 3 University Paris Diderot, Sorbonne Paris Cité, ECEVE, UMRS 1123, Paris, France; 4 AP-HP, Université Paris-VI, Institut de Cardiologie, Hôpital Pitié-Salpêtrière, Paris, France; Geisel School of Medicine at Dartmouth College, UNITED STATES

## Abstract

**Context:**

In France more than 70,000 deaths from diseases related to smoking are recorded each year, and since 2005 prevalence of tobacco has increased. Providing free access to smoking cessation treatment would reduce this burden. The aim of our study was to estimate the incremental cost-effectiveness ratios (ICER) of providing free access to cessation treatment taking into account the cost offsets associated with the reduction of the three main diseases related to smoking: lung cancer, chronic obstructive pulmonary disease (COPD) and cardiovascular disease (CVD). To measure the financial impact of such a measure we also conducted a probabilistic budget impact analysis.

**Methods and Findings:**

We performed a cost-effectiveness analysis using a Markov state-transition model that compared free access to cessation treatment to the existing coverage of €50 provided by the French statutory health insurance, taking into account the cost offsets among current French smokers aged 15–75 years. Our results were expressed by the incremental cost-effectiveness ratio in 2009 Euros per life year gained (LYG) at the lifetime horizon. We estimated a base case scenario and carried out a Monte Carlo sensitivity analysis to account for uncertainty. Assuming a participation rate of 7.3%, the ICER value for free access to cessation treatment was €3,868 per LYG in the base case. The variation of parameters provided a range of ICER values from -€736 to €15,715 per LYG. In 99% of cases, the ICER for full coverage was lower than €11,187 per LYG. The probabilistic budget impact analysis showed that the potential cost saving for lung cancer, COPD and CVD ranges from €15 million to €215 million at the five-year horizon for an initial cessation treatment cost of €125 million to €421 million.

**Conclusion:**

The results suggest that providing medical support to smokers in their attempts to quit is very cost-effective and may even result in cost savings.

## Introduction

The disastrous consequences of smoking in terms of morbidity and costs to the health care system have been clearly established for more than a dozen years [1,2]. However, the health actions that have been implemented are unequal to this burden. Each year more than 5 million deaths from smoking-related diseases are recorded worldwide [3]. Among them, three major diseases are responsible for more than half of the deaths: lung cancer, chronic obstructive pulmonary disease (COPD) and cardiovascular disease (CVD) [4]. France, with more than 70,000 smoking-related deaths annually, has not been spared this burden. Indeed, among the countries of western Europe and north America, France has the highest smoking prevalence [5], which after more than 25 years of decline increased again between 2005 and 2010 [6]. The costs related to the treatment of smoking-related diseases are almost fully covered by statutory health insurance (SHI) through a dedicated scheme for long-term chronic illnesses (*affections de longue durée*; ALD). In 2007, these costs accounted for 2.57% of the SHI annual budget (€3.3 billion) [7]. As the prevalence of lung cancer, COPD and CVD has steadily increased in France since 2005 [8], we can anticipate that health care costs related to these diseases will increase proportionally.

Controlling health expenditure is one of the major challenges facing health systems, and thus reducing the prevalence of diseases that are at least partly preventable constitutes an essential element in cost control. The scientific evidence on the effectiveness of providing free access to smoking cessation treatment has already been demonstrated [9–11]. This strategy ensures an increased number of cessation attempts and thus a higher ultimate successful quit rate [12–14]. Moreover, the medical management of smoking cessation is considered among the most cost-effective means of avoiding expenditures associated with the three main diseases [15–17].

To date, public funding for smoking cessation has been limited in France as in other countries. In 2007, an initiative to reduce the economic barrier to access cessation treatment was put into place in the form of a fixed annual SHI coverage of €50 per insured for expenditures related to two smoking cessation drugs. However, this measure has had no demonstrable effect as the prevalence of smoking and expenditures for smoking-related diseases have both continued to steadily increase [6], leading us to examine whether full coverage would be a cost-effective means of reducing smoking prevalence and related preventable diseases.

Previous studies have evaluated the cost effectiveness of full coverage of smoking cessation [18,19]. However, these studies did not take into account cost offsets for major diseases avoided nor did they estimate the budget impact of implementing such a policy. Our objective was to explore the potential effect of free access to smoking cessation treatment under the French SHI on the economic burden of smoking-related diseases. To do so, we estimated the incremental cost-effectiveness ratios (ICERs) of full coverage of smoking cessation compared to the fixed annual SHI coverage of €50 taking into account the cost offsets associated with the reduction of lung cancer, COPD and CVD and also measured the budget impact of this preventive action.

## Materials and Methods

### Study design

We performed a cost-effectiveness analysis on a fictive cohort of 1 000 smokers comparing full coverage of the medical management of smoking cessation to the existing €50 coverage by the French SHI, taking into account the potential cost offsets due to reduced incidence of lung cancer, COPD and CVD.

The target population was French smokers (defined as ≥1 cigarette/day) aged 15–75 years. Our fictive cohort was representative of the age and gender distribution of the target population [20,21]. Cost and health benefits were modeled over the lifetime of the cohort with a 3% discount rate [22]. The cost perspective was the SHI, and for reasons of data availability the base year of analysis of costs was 2009.

In order to estimate the cost effectiveness of full coverage, we chose the incremental cost-effectiveness ratio (ICER), which measures the cost per life-year gained due to the intervention. The ratio denominator is the measured gain in health expressed in years of life gained, and the ratio numerator is the cost associated with the health gain. The results were given in Euros per life-year gained (LYG) at the lifetime horizon for the entire cohort and by age and gender.

Due to some model parameter uncertainty we developed a base case scenario using the most plausible assumptions and then undertook deterministic and probabilistic sensitivity analyses.

### Strategies

We compared full coverage of a medically managed smoking cessation program to the existing €50 coverage provided by SHI. Despite the existence of guidelines for smoking cessation in France [23], there is no standard practice [24]. Thus, we based the proposed intervention on current practice in hospital-based outpatient clinics [25,26]. Treatment included prescription drugs available for smoking cessation treatment (oral and transdermal nicotine replacement therapy, varenicline and bupropion) combined with six consultations with doctors [19].

The comparator was the existing lump sum coverage of €50 for oral or transdermal nicotine replacement therapy prescribed during a consultation with a general practitioner.

### Model method

**Fig 1:**
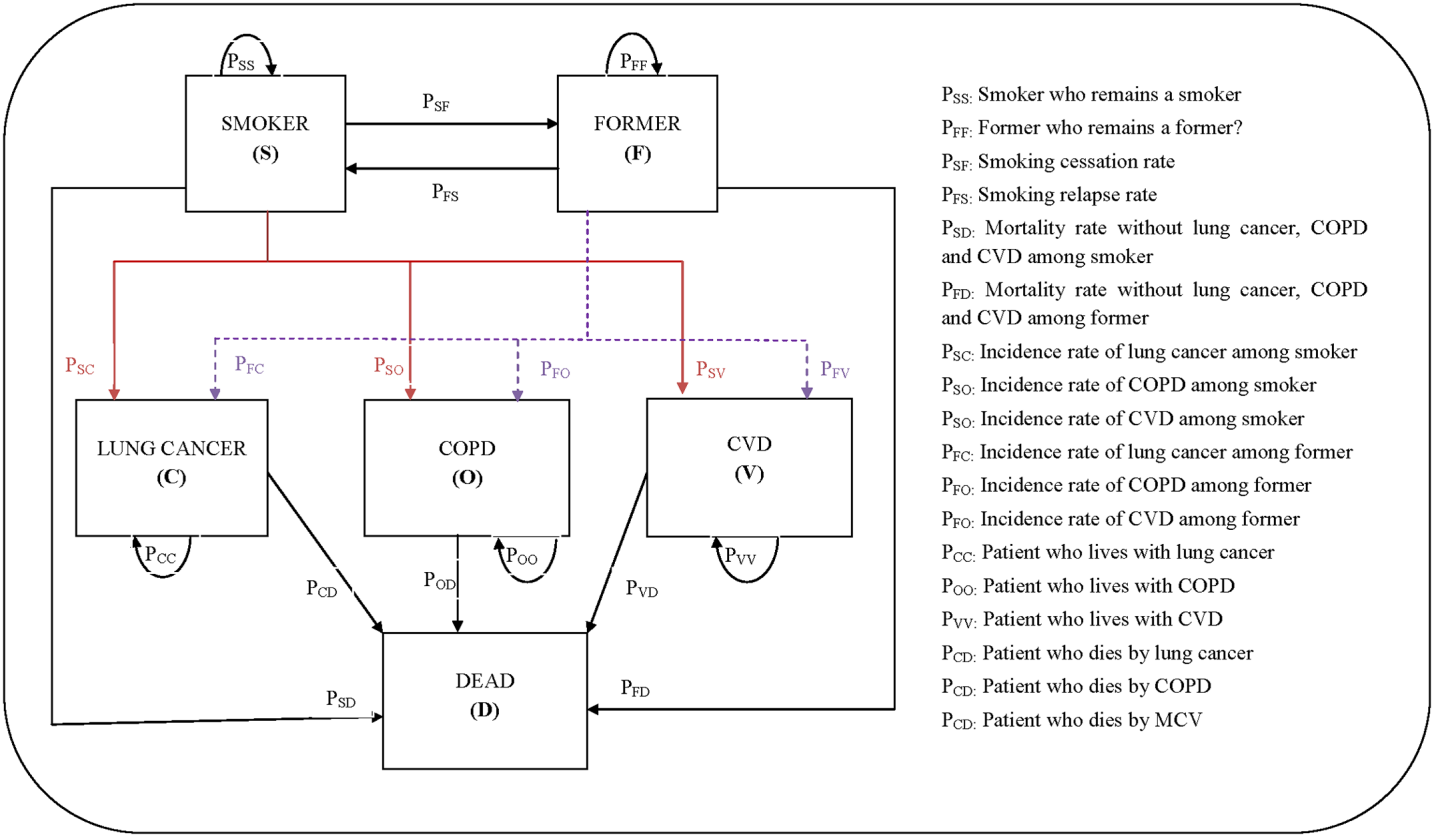
Schematic diagram of Markov model estimating effectiveness of smoking cessation strategies.

We used a Markov simulation model to describe the natural history of smokers until death [27]. The model consisted of six mutually exclusive states: smoker (S), former smoker (F), diagnosed with either lung cancer (C), COPD (O) or CVD such as stroke or coronary artery disease (V) and dead (D) (Fig 1). We used a one-year cycle. In the first cycle, all individuals are considered to be smokers. At the end of each cycle, smokers may be found in one of the following six states: remain smokers (P_SS_), stop smoking and become formers smokers (P_SF_), be diagnosed with one of three smoking related diseases (P_SC_, P_SO_, P_SV_) or die (P_SD_). From cycle two until the final lifetime cycle in the model, former smokers have five possibilities: they may remain former smokers (P_FF_), experience a relapse and become smokers again (P_FS_), be diagnosed with one of three smoking related diseases (P_FC_, P_FO_, P_FV_) or die (P_FD_). The dead state is an absorbing state (P_DD_), and the model stops when all individuals in the cohort are found in this state. The model was operated on Microsoft Office Excel 2007.

### Effectiveness

#### Cessation and relapse rates

The definitive cessation rate for the full coverage strategy was estimated at 7.04% in the base case with a 95% confidence interval [5.64%; 9.47%], as described in a previous study [19]. We relied on the natural cessation rate of 2.6% to which we applied an odds ratio of 1.95 based on results of a meta-analysis of randomized controlled trials of smoking cessation [28,29] and a second odds ratio of 1.39 based a meta-analysis of individual counseling [30]. No relapse rate was directly introduced into the model.

Given that smoking prevalence increased between 2005 and 2010 [6] while the number of individuals benefitting from the lump sum coverage of smoking cessation drugs between 2008 and 2013 decreased from 488,441 to 212,491 [31], we believe it is reasonable to assume that the existing €50 coverage has had no significant long-term impact on smoking behaviour. Thus we applied the natural cessation rate of 2.6% in our model.

#### Participation rate and number and frequency of cessation attempts covered

Our starting point in estimating the participation rate is based on the fact that 73% of smokers report that they would like to quit [32]. We consulted experts from French hospital-based outpatient clinics who estimated that only 10% who would like to quit are really motivated to quit, and we therefore applied a participation rate of 7.3% for the full coverage strategy in the base case, which corresponds to 10% of the 73% of smokers who are truly motivated to quit. This estimation is confirmed by data from the UK, where a similar medically managed smoking cessation program (the NHS Stop Smoking Services) has been in place since 1999, with reported participation of around 7% [33]. Because this parameter is very uncertain, we varied the estimate from 5% to 73%.

Based on data from French hospital-based outpatient clinics showing that an average of four attempts is necessary for a smoker who is fully supported both medically and psychologically to quit smoking (unpublished data), we chose four as the number of attempts fully covered by SHI in the model for the base case, given the limited resources of the public payer. Moreover, a relapsed smoker needs time between two consecutive quit attempts, and thus we assumed a frequency of every two years in the base case.

#### Incidence rate

There are no disease-specific annual incidence data on smokers (P_SC_, P_SO_, P_SV_) and former smokers (P_FC_, P_FO_, P_FV_) available in France. To estimate incidence rates of lung cancer, COPD and CVD we used the disease-specific mortality rates of each age group stratified by gender. This constitutes a conservative approach because the general mortality for both COPD and CVD is lower than their incidence [34,35], and in the case of lung cancer for which remission is extremely rare, mortality and incidence are nearly equivalent [36].

For smokers and non-smokers, we used the disease-specific mortality data for the three diseases from the Doll cohort in the UK (1971–1991) [1]. Because these data are not based on a French cohort and because the timeframe is slightly different, we made one spatial and one time adjustment. From a study of smoking-related mortality in developed countries [4], we obtained the mortality rate for each disease (lung cancer, COPD and vascular disease) for France and the UK for the years 1995 and 2000. We calculated ratios of the mortality data from France and the UK in 1995 for each of the diseases and applied them to the mortality data from the Doll cohort [1]. Next, we calculated ratios of the mortality data from France for each disease in 1995 and 2000 and applied them to the previously adjusted mortality data to obtain the most recent mortality data.

Since the Doll study [1] did not provide the mortality rate by disease and duration of cessation, we used the relative risk between non-smokers and former smokers by duration of cessation for the main smoking-related diseases found in a cancer prevention study (CPS1) [37]. We applied these relative risks to the time- and spatially-adjusted mortality rates for the three smoking-related diseases to obtain the mortality rates used in the model for non-smokers, smokers and former smokers by duration of cessation. Finally, given that the Doll cohort is exclusively male, we applied an estimate based on the actual lung cancer death ratio between men and women [38] to assess mortality data for women. This approach is justified because the consequences in terms of health are the same for men and women [39]. The data sources and the mortality data estimated by our method are shown in detail in S1–S4 Tables.

#### Mortality rate

To estimate the mortality rates for individuals diagnosed with lung cancer, COPD or CVD we used the life expectancy after diagnosis for each disease. In the case of lung cancer, we assumed that life expectancy at the time of diagnosis is independent of age and smoking status and dependent only on the type of cancer. Because life expectancy with lung cancer is around two years [40], we used a life expectancy of two years to estimate the mortality rate for lung cancer in our model. The life expectancy for individuals diagnosed with COPD is dependent on age and smoking status. We calculated this life expectancy by using the results of a multivariable Cox proportional hazard regression model between smokers, former smokers and non-smokers [41]. As with COPD, the life expectancy for individuals diagnosed with CVD is dependent on age and also partly on smoking status [42–44]. Since all survival curves are stratified on age, we used age as the sole parameter for the estimation of CVD life expectancy in the model. To estimate life expectancy following diagnosis of CVD we used the survival rates of two major cardiovascular diseases, first stroke event [42,44] and coronary artery disease [43], weighted by the contribution of each disease to total cardiovascular mortality. We ensured that survival time after a stroke or coronary artery disease was similar to the survival time of any CVD [8]. The general mortality rates for smokers (P_SD_) and former smokers (P_FD_) were calculated by subtracting the disease-specific mortality rates for lung cancer, COPD and CVD from the global mortality rate.

All effectiveness parameters are shown in Table 1.

**Table 1:** Effectiveness parameters of the model.

Parameters	Value	Uncertainty	Source
**Participation rate**			
Full coverage	7.30%	[5%,73%]	[19]–Expert
€50 coverage	3.75%	Fixed	
**Number of attempts covered**	4	2 or 6	[32]
**Frequency of attempts**	Biennial	annual or quadrennial	Expert
**Cessation rate**			
Full coverage	7.04%	CI95: [5.64%; 9.47%]	[21]
€50 coverage	2.60%		[61]
**Relapse rate**	Included in cessation rate	NA	
**Incidence rate**			[1,4,37]
Lung cancer	Life table		
COPD	Life table		
CVD	Life table		
**Mortality rate**			
**Lung cancer**			
All ages	2 years	External validation	[40]
**COPD**		External validation	[41]
**Smokers**			
age < 70	18 years		
age = 70–79	10 years		
age ≥80	3 years		
**Former smokers**			
age < 70	20 years		
age = 70–79	15 years		
age ≥80	5 years		
**Non-smokers**			
age < 80	20 years		
age ≥80	5 years		
**CVD**		External validation	[42–44]
age < 65	15 years		
age = 65–74	6 years		
age = 75–84	3 years		
age ≥85	1 years		

### Cost estimates

#### Intervention cost

For both strategies, the total cost included the costs associated with medical consultations and those associated with smoking cessation drugs. The total cost of full coverage was €333 for the first cycle, €132 for GP consultations and €201 for drugs [19] in the base case. The cost of the existing SHI coverage was €64.4 for the first cycle: €50 for drugs and €14.4 for a single doctor visit to obtain the prescription [19].

#### Cost offsets

Cost offsets were calculated based on the number of patients diagnosed with lung cancer, COPD or CVD in each strategy. We calculated the chronic illness cost per person in 2009 for each of the three diseases by taking the average total health care cost per person in 2009 for a chronic illness and subtracting the average total health care cost of individuals who did not have one or more covered chronic diseases [45].

#### Inflation

The annual inflation rate was calculated from the specific health care price indexes. From 2004 to 2008, the annual inflation rate was 0.23% for GP visits, 2.19% for drugs and -1.5% for chronic diseases in the base case [46].

The cost parameters are shown in Table 2.

**Table 2:** Cost parameters of the model.

Parameters	Value	Uncertainty	Source
**Cost of strategies**			[19]
Full coverage			
Doctors	€132		
Drugs	€201	[€120- €220]	
€50 coverage			
Doctors	€14	Fixed	
Drugs	€50	Fixed	
**Cost offset**			[45]
Lung cancer	€13 872	Fixed	
COPD	€6 562	Fixed	
CVD	€7 976	Fixed	
**Inflation rate**			[46]
GP visits	+0.23%	1.32%	
Drugs	2.19%	-5.38%	
Chronic diseases	-1.50%	+1.50%	
**Discount rate**	3%	0% or 6%	[52]

#### Uncertainty

First we conducted a deterministic sensitivity analysis exclusively on the number of attempts covered because this parameter is clearly an important health policy issue. Next we conducted a Monte Carlo probabilistic sensitivity analysis using the Statscorer v1.4 (4.9 MB) plug-in for Microsoft Office Excel 2007 to assess the effect of uncertainty on the model results. A total of 1,000 simulations were run. We used a log normal distribution for the cessation rate and a triangular distribution for participation rate and the cost of smoking cessation treatment. We applied a discrete distribution for the number and frequency of attempts covered, discounting rates and inflation rates. All uncertainty parameters are shown in Tables 1 and 2.

#### Budget impact

We estimated the direct cost to SHI of full coverage against the existing €50 coverage for the first year of implementation. We also estimated the cost offsets for each of the three main smoking-related diseases over a time horizon of five years [47]. We performed a Monte Carlo probabilistic budget impact analysis with the same distribution of parameters as in the ICER analyses. Because some policies are evaluated over a much longer term, we also provided the cost offset for each of the three main diseases over a time horizon of 10 and 20 years under the base case scenario.

#### External validation

We tested the external validity of our Markov model in two ways. First, we compared certain outputs generated by the model to those observed in the general population. We compared the life expectancy of the general French population in 2000 [48] and the time onset of chronic illness [8] in the general population generated by the model. Second, we compared the results of our model to a similar Markov model study conducted in Australia [49] also in addition to the actual results of the Stop Smoking Service, a fully covered smoking cessation program already implemented by the NHS in the UK [50]. We incorporated the specific parameters of both cases (cessation rate, participation rate, number and frequency of attempts) into our model to test external validity.

## Results

### Base case scenario

Under the base case scenario, assuming a participation rate of 7.3%, coverage of four cessation attempts by smokers every two years and a discount rate of 3%, the ICER value for full coverage of medically-managed smoking cessation was €3,868 per life year gained (LYG) at the lifetime horizon. The full coverage strategy resulted in the avoidance of 0.34 deaths per 1 000 population from lung cancer, COPD and CVD following four cessation attempts.

The ICER values varied by age and by gender (Table 3), and the full coverage strategy was more cost-effective for men than for women. For both genders the full coverage strategy was found to be most efficient for smokers aged 35 to 54.

**Table 3:** ICER per LYG stratified by age and gender in base case.

Age	Men	Women
15–24	€6,999	€8,391
25–34	€3,797	€4,642
35–44	€2,520	€3,138
45–54	€2,601	€3,056
55–64	€4,050	€4,345
65–74	€7,872	€7,551

### Sensitivity analysis

Using a deterministic approach, we varied the number of attempts covered, which resulted in ICER values of €3,745 per LYG (two covered attempts) and €4,022 per LYG (six covered attempts). With respect to life expectancy, full coverage resulted in a potential gain of 26.99 life years per 1 000 population (two covered attempts), 47.28 life years (four covered attempts) and 62.37 life years (six covered attempts) applying the discount rate of 0% only to the benefit.

**Fig 2:**
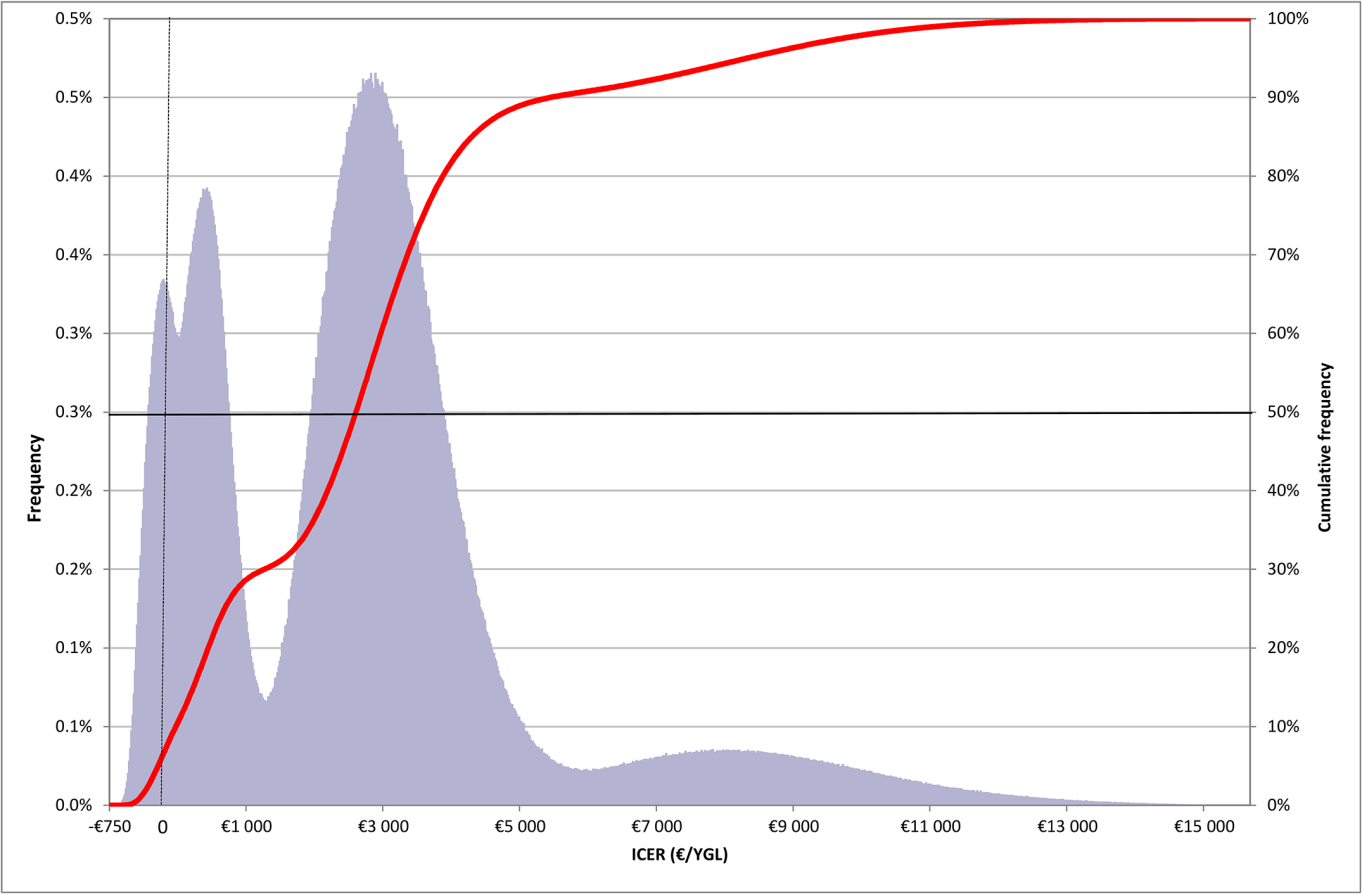
Probabilistic sensitivity analysis frequency and cumulative frequency of incremental cost-effectiveness ratio (ICER: incremental cost-effectiveness ratio; LYG: life-year gained).

Under the probabilistic approach, random variation of parameters provided a range of final ICER values from -€736 to €15,715 per LYG (Fig 2) and an average value of €3,034 per LYG (standard deviation = €2,447). The ICER values had a multivariate normal distribution. The cumulative distribution (curve, Fig 2) revealed that the cost-effectiveness ratio had a median value of €2,824 and an interquartile range of €914 to €3,812 per LYG. In 95% of cases, the ICER for full coverage was lower than €8,487 per LYG, and it was always less than €16,000 per LYG with cost savings in 5% of cases. These results demonstrate the robustness of our model because the random variation of key parameters had a moderate impact on the final ICER values.

### Budget impact

**Fig 3:**
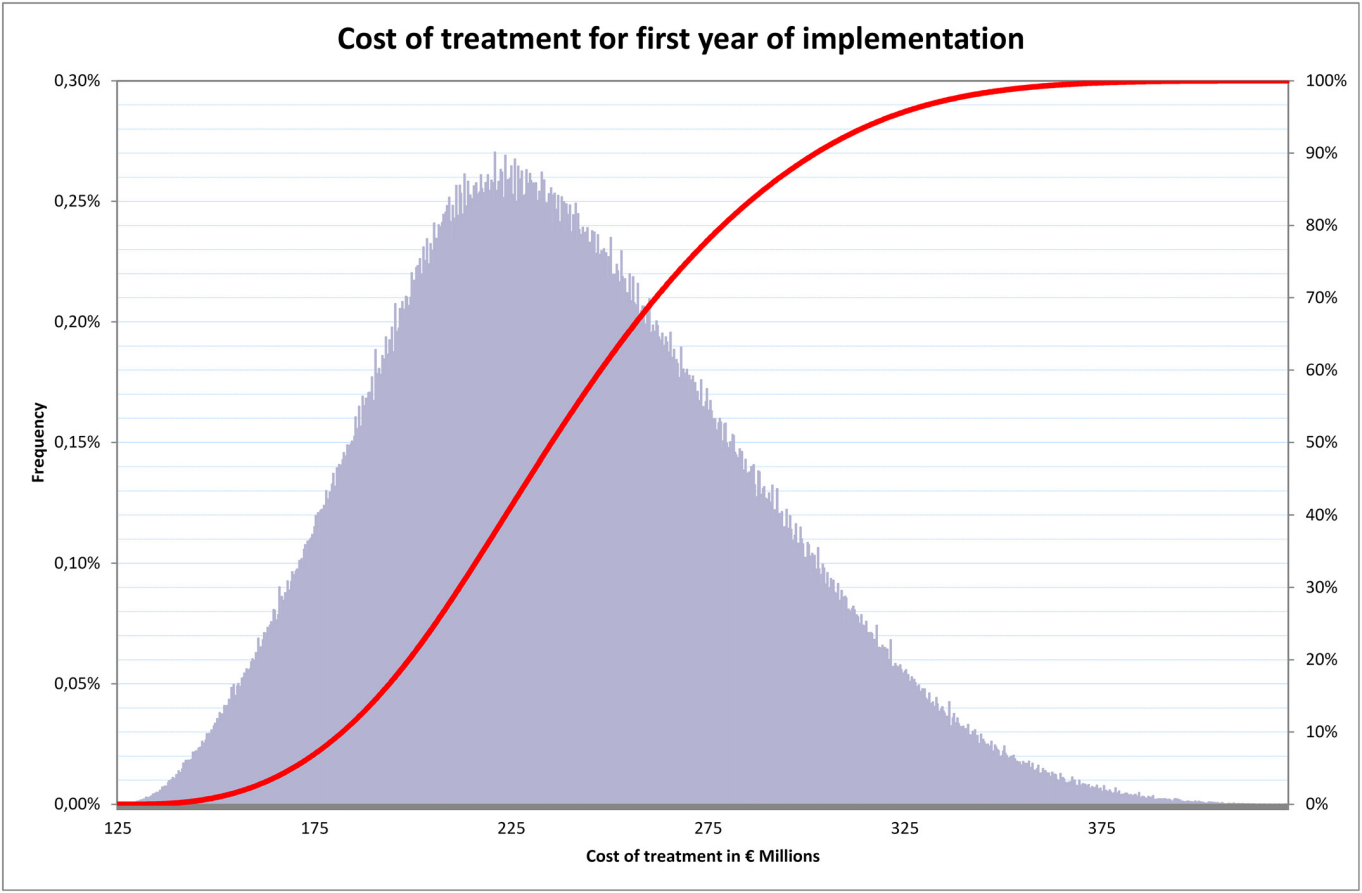
Probabilistic sensitivity analysis frequency and cumulative frequency of cost of cessation treatment.

**Fig 4:**
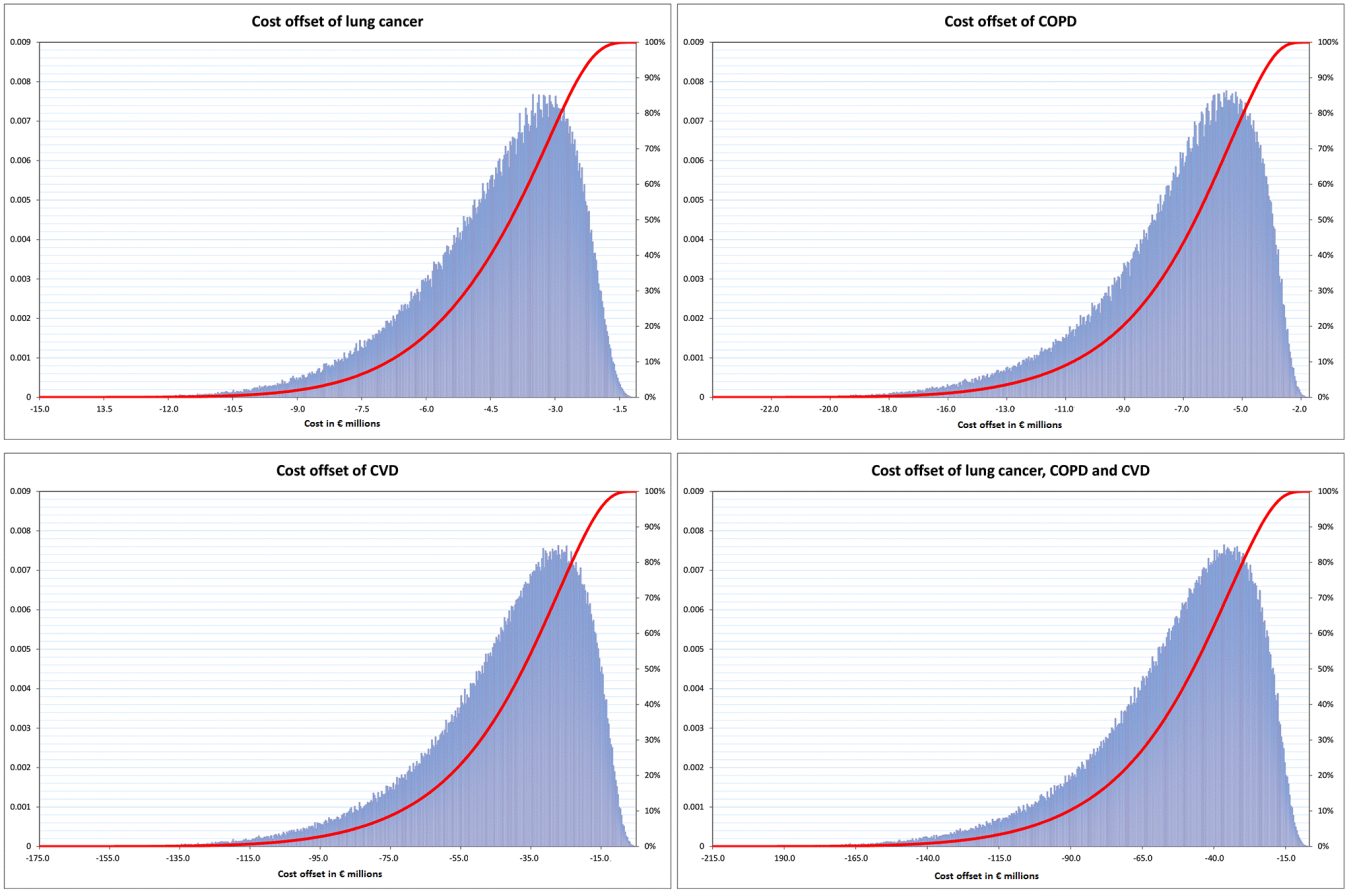
Probabilistic sensitivity analysis frequency and cumulative frequency of cost offsets for each of the three main smoking-related diseases over a time horizon of five years and the three combined.

When we performed a Monte Carlo probabilistic budget impact analysis of the cost for the first year of implementation (Fig 3), we estimated a mean cost of €240 million with a 95% confidence interval of €237 million to €243 million. In 95% of cases, the total medical cost for full coverage was less than €321 million. Fig 4 sets out the potential cost savings for lung cancer, COPD and CVD and the three diseases combined over a five-year horizon following the first year of implementation of the full coverage strategy in 2009. The mean potential cost offsets for SHI for lung cancer, COPD, CVD and the three combined were respectively €41 million, €6.9 million, €467 million and €57.8 million over five years. Under the base case, the longer term potential cost offsets for SHI for lung cancer, COPD and CVD were respectively €6.3 million, €9.5 million, €56.1 million over ten years and €14 million, €17.5 million, €89.6 million over twenty years.

### External validation

Table 4 shows that the life expectancy for smokers and non-smokers generated by the model at the age of 20 is close to that observed in the general population, with shorter life expectancy for smokers and longer for non-smokers. For both men and women, the difference in terms of life expectancy between lifelong smokers and lifelong non-smokers at the age of 20 is estimated by the model to be approximately 8 years. These results are close to those estimated for the Doll cohort [1]. The difference in life expectancy between lifelong smokers and the general population is only 4 years for men and more than 7 years for women, due to more tobacco-related deaths among men than women [4].

**Table 4:** Life expectancy in France at age 20 in the general population and as estimated by the model by smoking behavior.

Life expectancy at age 20			
	General population [48]	Smokers *(Estimated by the model)*	Non-smokers *(Estimated by the model)*
**Men**	56	52.15	60.6
**Women**	63.4	56.08	64

The times since onset of chronic illness by disease generated by the model are nearly equivalent to those in the general population for COPD and CVD based on SHI data with a time horizon of 20 years [8]. Concerning lung cancer, our model underestimated the time since onset of lung cancer compared to those observed (Table 5).

**Table 5:** Time since disease onset in chronic illness observed in France and generated by the model.

Time since disease onset							
		<1 year	1–2 years	3–5 years	5–9 years	10–15 years	>15 years
**Lung cancer**	Observed	28.5%	33.3%	13.0%	15.4%	6.4%	3.4%
	Modelling	50.0%	50.0%	-	-	-	-
**COPD**	Observed	9.5%	19.1%	13.9%	27.7%	15.8%	14.1%
	Modelling	9.8%	19.6%	13.2%	29.2%	19.0%	9.2%
**CVD**	Observed	10.0%	20.1%	15.1%	28.3%	13.5%	12.9%
	Modelling	10.4%	20.8%	15.2%	28.1%	25.6%	-

Using the same parameters as the Australian model cited in the method section [49], we estimated 42 life years gained and €549,000 in cost savings at the 10-year horizon for 1,000 quitters compared to the Australian estimate of 47 life years gained and cost savings of AUS$373,000 (€238,000 in €2009). By applying the UK participation and cessation rates to the French population in the model, the implementation of full coverage would save €159 million over 10 years in health care expenditures for lung cancer, COPD and CVD compared to ₤388 million (€543 million in €2009) in health care cost savings in the UK between 1996 and 2006 [50].

## Discussion

With an ICER value of €3,868 per life year gained for the base case scenario, our analysis demonstrated that full coverage of smoking cessation was a cost-effective strategy compared to the existing €50 coverage when cost offsets for lung cancer, COPD and CVD were included using a lifetime horizon. The full coverage strategy was most efficient for smokers aged 35 to 54. The cost for the first year of implementation was estimated at €240 million, and full coverage would save €57.6 million over a 5-year period in long-term chronic disease expenditures for the three primary smoking-related diseases at the five-year horizon

Although there are no official cost-effectiveness thresholds in France for decision making, our estimated ICER value is well below the standard thresholds used in western European countries, such as the UK, which has a cost-effectiveness threshold range of ₤20,000 to ₤30,000 per QALY. (€22,500 to €33,800 in €2009) [51]. According to the World Health Organization (WHO), a healthcare strategy is considered to be cost-effective if its ICER value is below one GDP per capita (€34,300 in France in 2009) [52]. Even when the more uncertain parameters of the model such as the cessation rate, the number of attempts covered or the discount rate were varied, full coverage of smoking cessation remained a very cost effective strategy at less than €16,000 per life year gained. Moreover, the full coverage strategy resulted in cost savings in 5% of cases.

The results of our study are consistent with previously published economic evaluations of smoking cessation interventions that account for cost offsets due to reduction of disease. In the UK, a similar Markov model [53] compared a dozen smoking cessation interventions to no intervention. The interventions closest to the strategy proposed in our study were dominant against no intervention. In terms of external validation of the model, we found that our results were very close to a similar Markov model study that simulated 1,000 quitters in Australia [49]. Our results were marginally higher in terms of potential cost savings because the annual costs of treating smoking-related disease are higher in France.

In comparison to countries in which full coverage of smoking cessation has been implemented such as the UK, our model also found savings in terms of health care expenditures, although of a smaller magnitude. The difference (€543 million for the UK versus €159 million in our model) is partly related to the fact that we only included lung cancer, COPD and CVD, which represent only 50% of the tobacco-related disease burden while the UK results are global.

We acknowledge some limitations regarding data availability and model structure. The accuracy of our modeling depends on the one hand how well the model structure describes the natural course of smoking in estimating outcomes and on the other hand the validity and accuracy of each of the model parameters. For that reason, we had to make certain assumptions. Except for the cessation rate and the survival rate for each assumption, we have systematically chosen the data that would avoid overestimating the results of the model.

We decided to base the cessation rate under the existing coverage on the natural cessation rate. Because there were no hypotheses for variation of this rate, we did not include it in probabilistic analysis. However, we compared free access to cessation treatment with no coverage at all, which resulted in an ICER value of €4,410 per LYG, which represents an increase of only 10% from the ICER value in the base case (€3,868 per LYG).

In estimating the survival rate with a CVD, we did not stratify the mortality rate on smoking history, which could have reduced the potential financial gain for free access to cessation treatment because ex-smokers live longer. However, a large body of research indicates that age has a greater impact on CVD life expectancy than the patient’s smoking status when both parameters are included in a multivariate model [42–44]. Moreover, there is conflicting evidence regarding the impact of smoking history on survival with CVD. Indeed, among the studies we used to estimate CVD mortality, there were mixed results in terms of the significance of smoking history: two found it to be partly significant (only for ex-smokers in one study and only for smokers compared to non-smokers in the other) [42,43], while the third study found smoking history was not significant [44].

The most significant limitation concerns the data used to estimate the incidence rates. We used mortality rate as a proxy to estimate the incidence rate of lung cancer, COPD and CVD. Given that incidence is lower than mortality for COPD and CVD and close to equivalent for lung cancer, we have underestimated the number of cases of these three diseases and thus their costs.

Because no data on mortality for smokers based on smoking status were available for France, we used data from a British database. Nonetheless, the smoking consequences are equivalent in France, and we applied ratios to adjust the mortality rates for each disease between France and UK in order to correct for spatial and time differences [4]. In the case of former smokers, in order to estimate their mortality rate by disease, we had to apply a relative risk ratio from the CPS I study for former smokers by disease to the disease-specific mortality rates of the Doll cohort. To ensure the validity of this approach, we conducted internal and external validity assessments. For internal validity we compared our estimated mortality rate to the aggregated results of the Doll study [54] and found the same results in terms of gain in life expectancy by age of smoking cessation. Our external validation showed that our assumptions and estimations of incidence rates and mortality rates were sufficiently robust to provide the data on life expectancy, time since disease onset and prevalence because they were close but never exceeded real life data observations. These results confirm the conservative nature of our approach.

Our method for estimating the aggregated cessation rate takes into account all available drug therapies and incorporates the relapse rate for each to obtain a definitive cessation rate. We confirmed that this method did not overestimate the cessation rate. Indeed, in the UK, the cessation rate at one year following implementation of its smoking cessation program was 14.6% [55]. To this cessation rate we applied the 37% [56] ten-year relapse rate after one year of cessation to estimate the lifetime relapse rate. In other words, 63% of the 14.6% of smokers who did not smoke for one year never relapse. This results in a definitive cessation rate of 9.2% (14.6% x 63%) which is higher than the 7.04% used in the model.

Concerning the structure of the model, in order to describe the natural course of smoking we limited ourselves to three major smoking-related diseases, while in reality there are more than fifty. We based this choice on the fact that these three diseases account for half of smoking-related morbidity [4] and because there is no data available on mortality by sex, age and smoking status for the other diseases. This conservative approach underestimates the savings due to smoking cessation.

In addition, our study is based on the current pricing of over-the-counter smoking cessation treatments. If these drugs were covered by SHI, the tariffs would be negotiated with pharmaceutical companies and a significant price reduction of 20% to 40% could be expected [57]. This would make full coverage even more cost-effective, with ICER values respectively of €1,115 and €1,360 per year of life gained and a reduction in the direct cost of full coverage with respective first-year budgets respectively of €200 million and €164 million compared to €235 million in the base case.

If we compare our study results to other health actions in cardiovascular prevention covered by SHI, the full coverage for smoking cessation would be the most cost-effective intervention with a relatively low annual budget impact. For example, the ICER of statins, the most commonly used drug for prevention of cardiovascular disease, ranges from €2,000 to over €300,000 per LYG depending on the level of risk [58]. Moreover, statins represent an annual budget outlay of more than €1 billion since 2009, an amount that has continued to increase due in part to inappropriate prescription to low risk patients [58].

If we compare the cost per patient for medically-managed smoking cessation with opiate substitution, we find a significant difference in the level of support. Indeed, with 500,000 opiate addicts in France [59] and an annual SHI expenditure of €100 million for drug substitution [60], the cost per patient amounts to €200 per year. By comparison, in 2009 approximately 500,000 individuals had recourse to the €50 coverage [31] for medical assistance to stop smoking, at an annual cost of €2 per smoker. Full coverage of smoking cessation would represent an annual cost per smoker of around €8.5, which is still far below what is spent on opiate substitution.

## Conclusion

We estimated the long-term cost-effectiveness of the full coverage of smoking cessation treatment from the SHI perspective in France, including cost offsets for the top three smoking-related diseases. The results suggest that providing free access to medical support to smokers in their attempts to quit is very cost effective and may even result in cost savings.

## Supporting Information

S1 Table (DOCX)

S2 Table (DOCX)

S3 Table (DOCX)

S4 Table (DOCX)
