# Supplementary material for: Cost Effectiveness of Free Access to Smoking Cessation Treatment in France Considering the Economic Burden of Smoking-Related Diseases
Source: PLoS One. 2016 Feb 24;11(2):e0148750. doi: 10.1371/journal.pone.0148750 (PMC4766094; doi:10.1371/journal.pone.0148750)
Supplement: S1 Table — (DOCX) [file pone.0148750.s001.docx]

S1 Table: Overview of adjusted mortality rate from lung cancer used in the model

| **Annual mortality rate(per 1000) by lung cancer stratified by age, gender and time since cessation** | | | | | | | | | | | | | | |
| --- | --- | --- | --- | --- | --- | --- | --- | --- | --- | --- | --- | --- | --- | --- |
| **Age** | **Smoker** | | **Former aged 15-24** | | **Former aged 25-34** | | **Former aged 35-44** | | **Former aged 45-54** | | **Former aged 55-64** | | **Former aged 65-74** | |
|  | M | F | M | F | M | F | M | F | M | F | M | F | M | F |
| From 15 to 34 | 0.0000 | 0.0000 | 0.0000 | 0.0000 | 0.0000 | 0.0000 | 0.0000 | 0.0000 | 0.0000 | 0.0000 | 0.0000 | 0.0000 | 0.0000 | 0.0000 |
| 35 | 0.0700 | 0.0525 | 0.0340 | 0.0255 | 0.0700 | 0.0525 | 0.0700 | 0.0525 | 0.0700 | 0.0525 | 0.0700 | 0.0525 | 0.0700 | 0.0525 |
| 36 | 0.0700 | 0.0525 | 0.0340 | 0.0255 | 0.0700 | 0.0525 | 0.0700 | 0.0525 | 0.0700 | 0.0525 | 0.0700 | 0.0525 | 0.0700 | 0.0525 |
| 37 | 0.0700 | 0.0525 | 0.0340 | 0.0255 | 0.0700 | 0.0525 | 0.0700 | 0.0525 | 0.0700 | 0.0525 | 0.0700 | 0.0525 | 0.0700 | 0.0525 |
| 38 | 0.0700 | 0.0525 | 0.0340 | 0.0255 | 0.0700 | 0.0525 | 0.0700 | 0.0525 | 0.0700 | 0.0525 | 0.0700 | 0.0525 | 0.0700 | 0.0525 |
| 39 | 0.0700 | 0.0525 | 0.0340 | 0.0255 | 0.0340 | 0.0255 | 0.0700 | 0.0525 | 0.0700 | 0.0525 | 0.0700 | 0.0525 | 0.0700 | 0.0525 |
| 40 | 0.4500 | 0.3375 | 0.0340 | 0.0255 | 0.0340 | 0.0255 | 0.4461 | 0.3346 | 0.4500 | 0.3375 | 0.4500 | 0.3375 | 0.4500 | 0.3375 |
| 41 | 0.4500 | 0.3375 | 0.0340 | 0.0255 | 0.0340 | 0.0255 | 0.4461 | 0.3346 | 0.4500 | 0.3375 | 0.4500 | 0.3375 | 0.4500 | 0.3375 |
| 42 | 0.4500 | 0.3375 | 0.0340 | 0.0255 | 0.0340 | 0.0255 | 0.4461 | 0.3346 | 0.4500 | 0.3375 | 0.4500 | 0.3375 | 0.4500 | 0.3375 |
| 43 | 0.4500 | 0.3375 | 0.0340 | 0.0255 | 0.0340 | 0.0255 | 0.2870 | 0.2152 | 0.4500 | 0.3375 | 0.4500 | 0.3375 | 0.4500 | 0.3375 |
| 44 | 0.4500 | 0.3375 | 0.0340 | 0.0255 | 0.0340 | 0.0255 | 0.2870 | 0.2152 | 0.4500 | 0.3375 | 0.4500 | 0.3375 | 0.4500 | 0.3375 |
| 45 | 1.0480 | 0.7860 | 0.0510 | 0.0383 | 0.0510 | 0.0383 | 0.4304 | 0.3228 | 1.0480 | 0.7860 | 1.0480 | 0.7860 | 1.0480 | 0.7860 |
| 46 | 1.0480 | 0.7860 | 0.0510 | 0.0383 | 0.0510 | 0.0383 | 0.4304 | 0.3228 | 1.0480 | 0.7860 | 1.0480 | 0.7860 | 1.0480 | 0.7860 |
| 47 | 1.0480 | 0.7860 | 0.0510 | 0.0383 | 0.0510 | 0.0383 | 0.4304 | 0.3228 | 1.0480 | 0.7860 | 1.0480 | 0.7860 | 1.0480 | 0.7860 |
| 48 | 1.0480 | 0.7860 | 0.0510 | 0.0383 | 0.0510 | 0.0383 | 0.4304 | 0.3228 | 1.0480 | 0.7860 | 1.0480 | 0.7860 | 1.0480 | 0.7860 |
| 49 | 1.0480 | 0.7860 | 0.0510 | 0.0383 | 0.0510 | 0.0383 | 0.4304 | 0.3228 | 1.0480 | 0.7860 | 1.0480 | 0.7860 | 1.0480 | 0.7860 |
| 50 | 1.8020 | 1.3515 | 0.0650 | 0.0488 | 0.0650 | 0.0488 | 0.4304 | 0.3228 | 1.0480 | 0.7860 | 1.8020 | 1.3515 | 1.8020 | 1.3515 |
| 51 | 1.8020 | 1.3515 | 0.0650 | 0.0488 | 0.0650 | 0.0488 | 0.4304 | 0.3228 | 1.0480 | 0.7860 | 1.8020 | 1.3515 | 1.8020 | 1.3515 |
| 52 | 1.8020 | 1.3515 | 0.0650 | 0.0488 | 0.0650 | 0.0488 | 0.4304 | 0.3228 | 1.0480 | 0.7860 | 1.8020 | 1.3515 | 1.8020 | 1.3515 |
| 53 | 1.8020 | 1.3515 | 0.0650 | 0.0488 | 0.0650 | 0.0488 | 0.4304 | 0.3228 | 1.0480 | 0.7860 | 1.8020 | 1.3515 | 1.8020 | 1.3515 |
| 54 | 1.8020 | 1.3515 | 0.0650 | 0.0488 | 0.0650 | 0.0488 | 0.4304 | 0.3228 | 1.0480 | 0.7860 | 1.8020 | 1.3515 | 1.8020 | 1.3515 |
| 55 | 2.7340 | 2.0505 | 0.0650 | 0.0488 | 0.0650 | 0.0488 | 0.4304 | 0.3228 | 1.0480 | 0.7860 | 2.7340 | 2.0505 | 2.7340 | 2.0505 |
| 56 | 2.7340 | 2.0505 | 0.0650 | 0.0488 | 0.0650 | 0.0488 | 0.4304 | 0.3228 | 1.0480 | 0.7860 | 2.7340 | 2.0505 | 2.7340 | 2.0505 |
| 57 | 2.7340 | 2.0505 | 0.0650 | 0.0488 | 0.0650 | 0.0488 | 0.4304 | 0.3228 | 1.0480 | 0.7860 | 2.7340 | 2.0505 | 2.7340 | 2.0505 |
| 58 | 2.7340 | 2.0505 | 0.0650 | 0.0488 | 0.0650 | 0.0488 | 0.4304 | 0.3228 | 1.0480 | 0.7860 | 2.7340 | 2.0505 | 2.7340 | 2.0505 |
| 59 | 2.7340 | 2.0505 | 0.0650 | 0.0488 | 0.0650 | 0.0488 | 0.4304 | 0.3228 | 1.0480 | 0.7860 | 2.7340 | 2.0505 | 2.7340 | 2.0505 |
| 60 | 2.8300 | 2.1225 | 0.0650 | 0.0488 | 0.0650 | 0.0488 | 0.4304 | 0.3228 | 1.0480 | 0.7860 | 2.1800 | 1.6350 | 2.8300 | 2.1225 |
| 61 | 2.8300 | 2.1225 | 0.0650 | 0.0488 | 0.0650 | 0.0488 | 0.4304 | 0.3228 | 1.0480 | 0.7860 | 2.1800 | 1.6350 | 2.8300 | 2.1225 |
| 62 | 2.8300 | 2.1225 | 0.0650 | 0.0488 | 0.0650 | 0.0488 | 0.4304 | 0.3228 | 1.0480 | 0.7860 | 2.1800 | 1.6350 | 2.8300 | 2.1225 |
| 63 | 2.8300 | 2.1225 | 0.0650 | 0.0488 | 0.0650 | 0.0488 | 0.4304 | 0.3228 | 1.0480 | 0.7860 | 2.1800 | 1.6350 | 2.8300 | 2.1225 |
| 64 | 2.8300 | 2.1225 | 0.0650 | 0.0488 | 0.0650 | 0.0488 | 0.4304 | 0.3228 | 1.0480 | 0.7860 | 2.1800 | 1.6350 | 2.8300 | 2.1225 |
| 65 | 4.0400 | 3.0300 | 0.4133 | 0.3100 | 0.4133 | 0.3100 | 0.4919 | 0.3689 | 1.1945 | 0.8959 | 3.4885 | 2.6164 | 4.0400 | 3.0300 |
| 66 | 4.0400 | 3.0300 | 0.4133 | 0.3100 | 0.4133 | 0.3100 | 0.4919 | 0.3689 | 1.1945 | 0.8959 | 3.4885 | 2.6164 | 4.0400 | 3.0300 |
| 67 | 4.0400 | 3.0300 | 0.4133 | 0.3100 | 0.4133 | 0.3100 | 0.4919 | 0.3689 | 1.1945 | 0.8959 | 3.4885 | 2.6164 | 4.0400 | 3.0300 |
| 68 | 4.0400 | 3.0300 | 0.4133 | 0.3100 | 0.4133 | 0.3100 | 0.7605 | 0.5704 | 1.1945 | 0.8959 | 3.4885 | 2.6164 | 4.0400 | 3.0300 |
| 69 | 4.0400 | 3.0300 | 0.4133 | 0.3100 | 0.4133 | 0.3100 | 0.7605 | 0.5704 | 1.1945 | 0.8959 | 3.4885 | 2.6164 | 4.0400 | 3.0300 |
| 70 | 6.4500 | 4.8375 | 0.4133 | 0.3100 | 0.4133 | 0.3100 | 0.7605 | 0.5704 | 1.1945 | 0.8959 | 3.2260 | 2.4195 | 5.4229 | 4.0672 |
| 71 | 6.4500 | 4.8375 | 0.4133 | 0.3100 | 0.4133 | 0.3100 | 0.7605 | 0.5704 | 1.1945 | 0.8959 | 3.2260 | 2.4195 | 5.4229 | 4.0672 |
| 72 | 6.4500 | 4.8375 | 0.4133 | 0.3100 | 0.4133 | 0.3100 | 0.7605 | 0.5704 | 1.1945 | 0.8959 | 3.2260 | 2.4195 | 5.4229 | 4.0672 |
| 73 | 6.4500 | 4.8375 | 0.4133 | 0.3100 | 0.4133 | 0.3100 | 1.3144 | 0.9858 | 1.3144 | 0.9858 | 3.2260 | 2.4195 | 3.4885 | 2.6164 |
| 74 | 6.4500 | 4.8375 | 0.4133 | 0.3100 | 0.4133 | 0.3100 | 1.3144 | 0.9858 | 1.3144 | 0.9858 | 3.2260 | 2.4195 | 3.4885 | 2.6164 |
| 75 | 6.3500 | 4.7625 | 0.4133 | 0.3100 | 0.4133 | 0.3100 | 1.3144 | 0.9858 | 1.3144 | 0.9858 | 3.5560 | 2.6670 | 3.4885 | 2.6164 |
| 76 | 6.3500 | 4.7625 | 0.4133 | 0.3100 | 0.4133 | 0.3100 | 1.3144 | 0.9858 | 1.3144 | 0.9858 | 3.5560 | 2.6670 | 3.4885 | 2.6164 |
| 77 | 6.3500 | 4.7625 | 0.4133 | 0.3100 | 0.4133 | 0.3100 | 1.3144 | 0.9858 | 1.3144 | 0.9858 | 3.5560 | 2.6670 | 3.4885 | 2.6164 |
| 78 | 6.3500 | 4.7625 | 0.4133 | 0.3100 | 0.4133 | 0.3100 | 1.3144 | 0.9858 | 1.3144 | 0.9858 | 3.5560 | 2.6670 | 1.9055 | 1.4291 |
| 79 | 6.3500 | 4.7625 | 0.4133 | 0.3100 | 0.4133 | 0.3100 | 1.3144 | 0.9858 | 1.3144 | 0.9858 | 3.5560 | 2.6670 | 1.9055 | 1.4291 |
| 80 | 7.5700 | 5.6775 | 0.5500 | 0.4125 | 0.5500 | 0.4125 | 1.3144 | 0.9858 | 1.3144 | 0.9858 | 3.3990 | 2.5493 | 3.3990 | 2.5493 |
| 81 | 7.5700 | 5.6775 | 0.5500 | 0.4125 | 0.5500 | 0.4125 | 1.3144 | 0.9858 | 1.3144 | 0.9858 | 3.3990 | 2.5493 | 3.3990 | 2.5493 |
| 82 | 7.5700 | 5.6775 | 0.5500 | 0.4125 | 0.5500 | 0.4125 | 1.3144 | 0.9858 | 1.3144 | 0.9858 | 3.3990 | 2.5493 | 3.3990 | 2.5493 |
| 83 | 7.5700 | 5.6775 | 0.5500 | 0.4125 | 0.5500 | 0.4125 | 1.3144 | 0.9858 | 1.7490 | 1.3118 | 3.3990 | 2.5493 | 3.3990 | 2.5493 |
| 84 | 7.5700 | 5.6775 | 0.5500 | 0.4125 | 0.5500 | 0.4125 | 1.3144 | 0.9858 | 1.7490 | 1.3118 | 3.3990 | 2.5493 | 3.3990 | 2.5493 |
| 85 | 7.8200 | 5.8650 | 0.5500 | 0.4125 | 0.5500 | 0.4125 | 1.3144 | 0.9858 | 3.3990 | 2.5493 | 3.3990 | 2.5493 | 3.3990 | 2.5493 |
| 86 | 7.8200 | 5.8650 | 0.5500 | 0.4125 | 0.5500 | 0.4125 | 1.3144 | 0.9858 | 3.3990 | 2.5493 | 3.3990 | 2.5493 | 3.3990 | 2.5493 |
| 87 | 7.8200 | 5.8650 | 0.5500 | 0.4125 | 0.5500 | 0.4125 | 1.3144 | 0.9858 | 3.3990 | 2.5493 | 3.3990 | 2.5493 | 3.3990 | 2.5493 |
| 88 | 7.8200 | 5.8650 | 0.5500 | 0.4125 | 0.5500 | 0.4125 | 1.3144 | 0.9858 | 3.3990 | 2.5493 | 3.3990 | 2.5493 | 3.3990 | 2.5493 |
| 89 | 7.8200 | 5.8650 | 0.5500 | 0.4125 | 0.5500 | 0.4125 | 1.3144 | 0.9858 | 3.3990 | 2.5493 | 3.3990 | 2.5493 | 3.3990 | 2.5493 |
| 90 | 7.8200 | 5.8650 | 0.5500 | 0.4125 | 0.5500 | 0.4125 | 1.3144 | 0.9858 | 3.3990 | 2.5493 | 3.3990 | 2.5493 | 3.3990 | 2.5493 |
| 91 | 7.8200 | 5.8650 | 0.5500 | 0.4125 | 0.5500 | 0.4125 | 1.3144 | 0.9858 | 3.3990 | 2.5493 | 3.3990 | 2.5493 | 3.3990 | 2.5493 |
| 92 | 7.8200 | 5.8650 | 0.5500 | 0.4125 | 0.5500 | 0.4125 | 1.3144 | 0.9858 | 3.3990 | 2.5493 | 3.3990 | 2.5493 | 3.3990 | 2.5493 |
| 93 | 7.8200 | 5.8650 | 0.5500 | 0.4125 | 0.5500 | 0.4125 | 1.3144 | 0.9858 | 3.3990 | 2.5493 | 3.3990 | 2.5493 | 3.3990 | 2.5493 |
| 94 | 7.8200 | 5.8650 | 0.5500 | 0.4125 | 0.5500 | 0.4125 | 1.3144 | 0.9858 | 3.3990 | 2.5493 | 3.3990 | 2.5493 | 3.3990 | 2.5493 |
| 95 | 7.8200 | 5.8650 | 0.5500 | 0.4125 | 0.5500 | 0.4125 | 1.3144 | 0.9858 | 3.3990 | 2.5493 | 3.3990 | 2.5493 | 3.3990 | 2.5493 |
| 96 | 7.8200 | 5.8650 | 0.5500 | 0.4125 | 0.5500 | 0.4125 | 1.3144 | 0.9858 | 3.3990 | 2.5493 | 3.3990 | 2.5493 | 3.3990 | 2.5493 |
| 97 | 7.8200 | 5.8650 | 0.5500 | 0.4125 | 0.5500 | 0.4125 | 1.3144 | 0.9858 | 3.3990 | 2.5493 | 3.3990 | 2.5493 | 3.3990 | 2.5493 |
| 98 | 7.8200 | 5.8650 | 0.5500 | 0.4125 | 0.5500 | 0.4125 | 1.3144 | 0.9858 | 3.3990 | 2.5493 | 3.3990 | 2.5493 | 3.3990 | 2.5493 |
| 99 | 7.8200 | 5.8650 | 0.5500 | 0.4125 | 0.5500 | 0.4125 | 1.3144 | 0.9858 | 3.3990 | 2.5493 | 3.3990 | 2.5493 | 3.3990 | 2.5493 |
| 100 | 7.8200 | 5.8650 | 0.5500 | 0.4125 | 0.5500 | 0.4125 | 1.3144 | 0.9858 | 3.3990 | 2.5493 | 3.3990 | 2.5493 | 3.3990 | 2.5493 |

References:

1. Doll R, Peto R, Wheatley K, Gray R, Sutherland I (1994) Mortality in relation to smoking: 40 years’ observations on male British doctors. BMJ 309: 901–911. doi:10.1136/bmj.309.6959.901.

2. Rasmussen SR, Prescott E, Sørensen TI, Søgaard J (2004) The total lifetime costs of smoking. The European Journal of Public Health 14: 95–100.

3. WHO (n.d.) REPORT On THE global tobacoo epidemic, 2011. WHO. Available: http://whqlibdoc.who.int/publications/2011/9789240687813_eng.pdf?ua=1.

4. Peto R, Lopez AD, Boreham J, Thun M (2006) Mortality from smoking in developed countries 1950-2000 (2nd edition). Available: http://www.ctsu.ox.ac.uk/deathsfromsmoking/download%20files/Original%20research/Mortality%20from%20smoking%20in%20developed%20countries%201950-2000%20%282nd%20ed.%29.pdf.
