# Supplementary material for: Cost Effectiveness of Free Access to Smoking Cessation Treatment in France Considering the Economic Burden of Smoking-Related Diseases
Source: PLoS One. 2016 Feb 24;11(2):e0148750. doi: 10.1371/journal.pone.0148750 (PMC4766094; doi:10.1371/journal.pone.0148750)
Supplement: S2 Table — (DOCX) [file pone.0148750.s002.docx]

S2 Table: Overview of adjusted mortality rate from COPD used in the model

| **Annual mortality rate(per 1000) by COPD stratified by age, gender and time since cessation** | | | | | | | | | | | | | | |
| --- | --- | --- | --- | --- | --- | --- | --- | --- | --- | --- | --- | --- | --- | --- |
| **Age** | **Smoker** | | **Former aged 15-24** | | **Former aged 25-34** | | **Former aged 35-44** | | **Former aged 45-54** | | **Former aged 55-64** | | **Former aged 65-74** | |
|  | M | F | M | F | M | F | M | F | M | F | M | F | M | F |
| From  15 to 34 | 0.0000 | 0.0000 | 0.0000 | 0.0000 | 0.0000 | 0.0000 | 0.0000 | 0.0000 | 0.0000 | 0.0000 | 0.0000 | 0.0000 | 0.0000 | 0.0000 |
| 35 | 0.0160 | 0.0120 | 0.0070 | 0.0053 | 0.0160 | 0.0120 | 0.0160 | 0.0120 | 0.0160 | 0.0120 | 0.0160 | 0.0120 | 0.0160 | 0.0120 |
| 36 | 0.0160 | 0.0120 | 0.0070 | 0.0053 | 0.0160 | 0.0120 | 0.0160 | 0.0120 | 0.0160 | 0.0120 | 0.0160 | 0.0120 | 0.0160 | 0.0120 |
| 37 | 0.0160 | 0.0120 | 0.0070 | 0.0053 | 0.0160 | 0.0120 | 0.0160 | 0.0120 | 0.0160 | 0.0120 | 0.0160 | 0.0120 | 0.0160 | 0.0120 |
| 38 | 0.0160 | 0.0120 | 0.0070 | 0.0053 | 0.0160 | 0.0120 | 0.0160 | 0.0120 | 0.0160 | 0.0120 | 0.0160 | 0.0120 | 0.0160 | 0.0120 |
| 39 | 0.0160 | 0.0120 | 0.0070 | 0.0053 | 0.0070 | 0.0053 | 0.0160 | 0.0120 | 0.0160 | 0.0120 | 0.0160 | 0.0120 | 0.0160 | 0.0120 |
| 40 | 0.0280 | 0.0210 | 0.0070 | 0.0053 | 0.0070 | 0.0053 | 0.0280 | 0.0210 | 0.0280 | 0.0210 | 0.0280 | 0.0210 | 0.0280 | 0.0210 |
| 41 | 0.0280 | 0.0210 | 0.0070 | 0.0053 | 0.0070 | 0.0053 | 0.0280 | 0.0210 | 0.0280 | 0.0210 | 0.0280 | 0.0210 | 0.0280 | 0.0210 |
| 42 | 0.0280 | 0.0210 | 0.0070 | 0.0053 | 0.0070 | 0.0053 | 0.0280 | 0.0210 | 0.0280 | 0.0210 | 0.0280 | 0.0210 | 0.0280 | 0.0210 |
| 43 | 0.0280 | 0.0210 | 0.0070 | 0.0053 | 0.0070 | 0.0053 | 0.0280 | 0.0210 | 0.0280 | 0.0210 | 0.0280 | 0.0210 | 0.0280 | 0.0210 |
| 44 | 0.0280 | 0.0210 | 0.0070 | 0.0053 | 0.0070 | 0.0053 | 0.0280 | 0.0210 | 0.0280 | 0.0210 | 0.0280 | 0.0210 | 0.0280 | 0.0210 |
| 45 | 0.0660 | 0.0495 | 0.0130 | 0.0098 | 0.0130 | 0.0098 | 0.0660 | 0.0495 | 0.0660 | 0.0495 | 0.0660 | 0.0495 | 0.0660 | 0.0495 |
| 46 | 0.0660 | 0.0495 | 0.0130 | 0.0098 | 0.0130 | 0.0098 | 0.0660 | 0.0495 | 0.0660 | 0.0495 | 0.0660 | 0.0495 | 0.0660 | 0.0495 |
| 47 | 0.0660 | 0.0495 | 0.0130 | 0.0098 | 0.0130 | 0.0098 | 0.0660 | 0.0495 | 0.0660 | 0.0495 | 0.0660 | 0.0495 | 0.0660 | 0.0495 |
| 48 | 0.0660 | 0.0495 | 0.0130 | 0.0098 | 0.0130 | 0.0098 | 0.0660 | 0.0495 | 0.0660 | 0.0495 | 0.0660 | 0.0495 | 0.0660 | 0.0495 |
| 49 | 0.0660 | 0.0495 | 0.0130 | 0.0098 | 0.0130 | 0.0098 | 0.0660 | 0.0495 | 0.0660 | 0.0495 | 0.0660 | 0.0495 | 0.0660 | 0.0495 |
| 50 | 0.2530 | 0.1898 | 0.0170 | 0.0128 | 0.0170 | 0.0128 | 0.1756 | 0.1317 | 0.2530 | 0.1898 | 0.2530 | 0.1898 | 0.2530 | 0.1898 |
| 51 | 0.2530 | 0.1898 | 0.0170 | 0.0128 | 0.0170 | 0.0128 | 0.1756 | 0.1317 | 0.2530 | 0.1898 | 0.2530 | 0.1898 | 0.2530 | 0.1898 |
| 52 | 0.2530 | 0.1898 | 0.0170 | 0.0128 | 0.0170 | 0.0128 | 0.1756 | 0.1317 | 0.2530 | 0.1898 | 0.2530 | 0.1898 | 0.2530 | 0.1898 |
| 53 | 0.2530 | 0.1898 | 0.0170 | 0.0128 | 0.0170 | 0.0128 | 0.1756 | 0.1317 | 0.2530 | 0.1898 | 0.2530 | 0.1898 | 0.2530 | 0.1898 |
| 54 | 0.2530 | 0.1898 | 0.0170 | 0.0128 | 0.0170 | 0.0128 | 0.1756 | 0.1317 | 0.2530 | 0.1898 | 0.2530 | 0.1898 | 0.2530 | 0.1898 |
| 55 | 0.3010 | 0.2258 | 0.0170 | 0.0128 | 0.0170 | 0.0128 | 0.1756 | 0.1317 | 0.3010 | 0.2258 | 0.3010 | 0.2258 | 0.3010 | 0.2258 |
| 56 | 0.3010 | 0.2258 | 0.0170 | 0.0128 | 0.0170 | 0.0128 | 0.1756 | 0.1317 | 0.3010 | 0.2258 | 0.3010 | 0.2258 | 0.3010 | 0.2258 |
| 57 | 0.3010 | 0.2258 | 0.0170 | 0.0128 | 0.0170 | 0.0128 | 0.1756 | 0.1317 | 0.3010 | 0.2258 | 0.3010 | 0.2258 | 0.3010 | 0.2258 |
| 58 | 0.3010 | 0.2258 | 0.0170 | 0.0128 | 0.0170 | 0.0128 | 0.1756 | 0.1317 | 0.3010 | 0.2258 | 0.3010 | 0.2258 | 0.3010 | 0.2258 |
| 59 | 0.3010 | 0.2258 | 0.0170 | 0.0128 | 0.0170 | 0.0128 | 0.1756 | 0.1317 | 0.3010 | 0.2258 | 0.3010 | 0.2258 | 0.3010 | 0.2258 |
| 60 | 1.0860 | 0.8145 | 0.0170 | 0.0128 | 0.0170 | 0.0128 | 0.1756 | 0.1317 | 0.3010 | 0.2258 | 0.6144 | 0.4608 | 1.0860 | 0.8145 |
| 61 | 1.0860 | 0.8145 | 0.0170 | 0.0128 | 0.0170 | 0.0128 | 0.1756 | 0.1317 | 0.3010 | 0.2258 | 0.6144 | 0.4608 | 1.0860 | 0.8145 |
| 62 | 1.0860 | 0.8145 | 0.0170 | 0.0128 | 0.0170 | 0.0128 | 0.1756 | 0.1317 | 0.3010 | 0.2258 | 0.6144 | 0.4608 | 1.0860 | 0.8145 |
| 63 | 1.0860 | 0.8145 | 0.0170 | 0.0128 | 0.0170 | 0.0128 | 0.1756 | 0.1317 | 0.3010 | 0.2258 | 0.6144 | 0.4608 | 1.0860 | 0.8145 |
| 64 | 1.0860 | 0.8145 | 0.0170 | 0.0128 | 0.0170 | 0.0128 | 0.1756 | 0.1317 | 0.3010 | 0.2258 | 0.6144 | 0.4608 | 1.0860 | 0.8145 |
| 65 | 1.2260 | 0.9195 | 0.0170 | 0.0128 | 0.0170 | 0.0128 | 0.1756 | 0.1317 | 0.3010 | 0.2258 | 0.6144 | 0.4608 | 1.2260 | 0.9195 |
| 66 | 1.2260 | 0.9195 | 0.0170 | 0.0128 | 0.0170 | 0.0128 | 0.1756 | 0.1317 | 0.3010 | 0.2258 | 0.6144 | 0.4608 | 1.2260 | 0.9195 |
| 67 | 1.2260 | 0.9195 | 0.0170 | 0.0128 | 0.0170 | 0.0128 | 0.1756 | 0.1317 | 0.3010 | 0.2258 | 0.6144 | 0.4608 | 1.2260 | 0.9195 |
| 68 | 1.2260 | 0.9195 | 0.0170 | 0.0128 | 0.0170 | 0.0128 | 0.1756 | 0.1317 | 0.3010 | 0.2258 | 0.6144 | 0.4608 | 1.2260 | 0.9195 |
| 69 | 1.2260 | 0.9195 | 0.0170 | 0.0128 | 0.0170 | 0.0128 | 0.1756 | 0.1317 | 0.3010 | 0.2258 | 0.6144 | 0.4608 | 1.2260 | 0.9195 |
| 70 | 1.5960 | 1.1970 | 0.0320 | 0.0240 | 0.0320 | 0.0240 | 0.1756 | 0.1317 | 0.3010 | 0.2258 | 0.7980 | 0.5985 | 1.1565 | 0.8674 |
| 71 | 1.5960 | 1.1970 | 0.0320 | 0.0240 | 0.0320 | 0.0240 | 0.1756 | 0.1317 | 0.3010 | 0.2258 | 0.7980 | 0.5985 | 1.1565 | 0.8674 |
| 72 | 1.5960 | 1.1970 | 0.0320 | 0.0240 | 0.0320 | 0.0240 | 0.1756 | 0.1317 | 0.3010 | 0.2258 | 0.7980 | 0.5985 | 1.1565 | 0.8674 |
| 73 | 1.5960 | 1.1970 | 0.0320 | 0.0240 | 0.0320 | 0.0240 | 0.1756 | 0.1317 | 0.3010 | 0.2258 | 0.7980 | 0.5985 | 1.1565 | 0.8674 |
| 74 | 1.5960 | 1.1970 | 0.0320 | 0.0240 | 0.0320 | 0.0240 | 0.1756 | 0.1317 | 0.3010 | 0.2258 | 0.7980 | 0.5985 | 1.1565 | 0.8674 |
| 75 | 4.9140 | 3.6855 | 0.1460 | 0.1095 | 0.1460 | 0.1095 | 0.3635 | 0.2727 | 0.3010 | 0.2258 | 1.3000 | 0.9750 | 2.7579 | 2.0685 |
| 76 | 4.9140 | 3.6855 | 0.1460 | 0.1095 | 0.1460 | 0.1095 | 0.3635 | 0.2727 | 0.3010 | 0.2258 | 1.3000 | 0.9750 | 2.7579 | 2.0685 |
| 77 | 4.9140 | 3.6855 | 0.1460 | 0.1095 | 0.1460 | 0.1095 | 0.3635 | 0.2727 | 0.3010 | 0.2258 | 1.3000 | 0.9750 | 2.7579 | 2.0685 |
| 78 | 4.9140 | 3.6855 | 0.1460 | 0.1095 | 0.1460 | 0.1095 | 0.3635 | 0.2727 | 0.3329 | 0.2497 | 1.3000 | 0.9750 | 2.7579 | 2.0685 |
| 79 | 4.9140 | 3.6855 | 0.1460 | 0.1095 | 0.1460 | 0.1095 | 0.3635 | 0.2727 | 0.3329 | 0.2497 | 1.3000 | 0.9750 | 2.7579 | 2.0685 |
| 80 | 6.2080 | 4.6560 | 0.2610 | 0.1958 | 0.2610 | 0.1958 | 0.6499 | 0.4874 | 0.6499 | 0.4874 | 3.4890 | 2.6168 | 3.4890 | 2.6168 |
| 81 | 6.2080 | 4.6560 | 0.2610 | 0.1958 | 0.2610 | 0.1958 | 0.6499 | 0.4874 | 0.6499 | 0.4874 | 3.4890 | 2.6168 | 3.4890 | 2.6168 |
| 82 | 6.2080 | 4.6560 | 0.2610 | 0.1958 | 0.2610 | 0.1958 | 0.6499 | 0.4874 | 0.6499 | 0.4874 | 3.4890 | 2.6168 | 3.4890 | 2.6168 |
| 83 | 6.2080 | 4.6560 | 0.2610 | 0.1958 | 0.2610 | 0.1958 | 0.6499 | 0.4874 | 0.6499 | 0.4874 | 3.4890 | 2.6168 | 3.4890 | 2.6168 |
| 84 | 6.2080 | 4.6560 | 0.2610 | 0.1958 | 0.2610 | 0.1958 | 0.6499 | 0.4874 | 0.6499 | 0.4874 | 3.4890 | 2.6168 | 3.4890 | 2.6168 |
| 85 | 6.7950 | 5.0963 | 0.2540 | 0.1905 | 0.2540 | 0.1905 | 0.6325 | 0.4743 | 3.4890 | 2.6168 | 3.4890 | 2.6168 | 3.4890 | 2.6168 |
| 86 | 6.7950 | 5.0963 | 0.2540 | 0.1905 | 0.2540 | 0.1905 | 0.6325 | 0.4743 | 3.4890 | 2.6168 | 3.4890 | 2.6168 | 3.4890 | 2.6168 |
| 87 | 6.7950 | 5.0963 | 0.2540 | 0.1905 | 0.2540 | 0.1905 | 0.6325 | 0.4743 | 3.4890 | 2.6168 | 3.4890 | 2.6168 | 3.4890 | 2.6168 |
| 88 | 6.7950 | 5.0963 | 0.2540 | 0.1905 | 0.2540 | 0.1905 | 0.6325 | 0.4743 | 3.4890 | 2.6168 | 3.4890 | 2.6168 | 3.4890 | 2.6168 |
| 89 | 6.7950 | 5.0963 | 0.2540 | 0.1905 | 0.2540 | 0.1905 | 0.6325 | 0.4743 | 3.4890 | 2.6168 | 3.4890 | 2.6168 | 3.4890 | 2.6168 |
| 90 | 6.7950 | 5.0963 | 0.2540 | 0.1905 | 0.2540 | 0.1905 | 0.6325 | 0.4743 | 3.4890 | 2.6168 | 3.4890 | 2.6168 | 3.4890 | 2.6168 |
| 91 | 6.7950 | 5.0963 | 0.2540 | 0.1905 | 0.2540 | 0.1905 | 0.6325 | 0.4743 | 3.4890 | 2.6168 | 3.4890 | 2.6168 | 3.4890 | 2.6168 |
| 92 | 6.7950 | 5.0963 | 0.2540 | 0.1905 | 0.2540 | 0.1905 | 0.6325 | 0.4743 | 3.4890 | 2.6168 | 3.4890 | 2.6168 | 3.4890 | 2.6168 |
| 93 | 6.7950 | 5.0963 | 0.2540 | 0.1905 | 0.2540 | 0.1905 | 0.6325 | 0.4743 | 3.4890 | 2.6168 | 3.4890 | 2.6168 | 3.4890 | 2.6168 |
| 94 | 6.7950 | 5.0963 | 0.2540 | 0.1905 | 0.2540 | 0.1905 | 0.6325 | 0.4743 | 3.4890 | 2.6168 | 3.4890 | 2.6168 | 3.4890 | 2.6168 |
| 95 | 6.7950 | 5.0963 | 0.2540 | 0.1905 | 0.2540 | 0.1905 | 0.6325 | 0.4743 | 3.4890 | 2.6168 | 3.4890 | 2.6168 | 3.4890 | 2.6168 |
| 96 | 6.7950 | 5.0963 | 0.2540 | 0.1905 | 0.2540 | 0.1905 | 0.6325 | 0.4743 | 3.4890 | 2.6168 | 3.4890 | 2.6168 | 3.4890 | 2.6168 |
| 97 | 6.7950 | 5.0963 | 0.2540 | 0.1905 | 0.2540 | 0.1905 | 0.6325 | 0.4743 | 3.4890 | 2.6168 | 3.4890 | 2.6168 | 3.4890 | 2.6168 |
| 98 | 6.7950 | 5.0963 | 0.2540 | 0.1905 | 0.2540 | 0.1905 | 0.6325 | 0.4743 | 3.4890 | 2.6168 | 3.4890 | 2.6168 | 3.4890 | 2.6168 |
| 99 | 6.7950 | 5.0963 | 0.2540 | 0.1905 | 0.2540 | 0.1905 | 0.6325 | 0.4743 | 3.4890 | 2.6168 | 3.4890 | 2.6168 | 3.4890 | 2.6168 |
| 100 | 6.7950 | 5.0963 | 0.2540 | 0.1905 | 0.2540 | 0.1905 | 0.6325 | 0.4743 | 3.4890 | 2.6168 | 3.4890 | 2.6168 | 3.4890 | 2.6168 |

References:

1. Doll R, Peto R, Wheatley K, Gray R, Sutherland I (1994) Mortality in relation to smoking: 40 years’ observations on male British doctors. BMJ 309: 901–911. doi:10.1136/bmj.309.6959.901.

2. Rasmussen SR, Prescott E, Sørensen TI, Søgaard J (2004) The total lifetime costs of smoking. The European Journal of Public Health 14: 95–100.

3. WHO (n.d.) REPORT On THE global tobacoo epidemic, 2011. WHO. Available: http://whqlibdoc.who.int/publications/2011/9789240687813_eng.pdf?ua=1.

4. Peto R, Lopez AD, Boreham J, Thun M (2006) Mortality from smoking in developed countries 1950-2000 (2nd edition). Available: http://www.ctsu.ox.ac.uk/deathsfromsmoking/download%20files/Original%20research/Mortality%20from%20smoking%20in%20developed%20countries%201950-2000%20%282nd%20ed.%29.pdf.
