# Supplementary material for: Cost Effectiveness of Free Access to Smoking Cessation Treatment in France Considering the Economic Burden of Smoking-Related Diseases
Source: PLoS One. 2016 Feb 24;11(2):e0148750. doi: 10.1371/journal.pone.0148750 (PMC4766094; doi:10.1371/journal.pone.0148750)
Supplement: S3 Table — (DOCX) [file pone.0148750.s003.docx]

S3 Table: Overview of adjusted mortality rate from CVD used in the model

| **Annual mortality rate(per 1000) by CVD stratified by age, gender and time since cessation** | | | | | | | | | | | | | | |
| --- | --- | --- | --- | --- | --- | --- | --- | --- | --- | --- | --- | --- | --- | --- |
| **Age** | **Smoker** | | **Former aged 15-24** | | **Former aged 25-34** | | **Former aged 35-44** | | **Former aged 45-54** | | **Former aged 55-64** | | **Former aged 65-74** | |
|  | M | F | M | F | M | F | M | F | M | F | M | F | M | F |
| From  15 to 34 | 0.0000 | 0.0000 | 0.0000 | 0.0000 | 0.0000 | 0.0000 | 0.0000 | 0.0000 | 0.0000 | 0.0000 | 0.0000 | 0.0000 | 0.0000 | 0.0000 |
| 35 | 0.4553 | 0.3415 | 0.2942 | 0.2206 | 0.1850 | 0.1388 | 0.4553 | 0.3415 | 0.4553 | 0.3415 | 0.4553 | 0.3415 | 0.4553 | 0.3415 |
| 36 | 0.4553 | 0.3415 | 0.2942 | 0.2206 | 0.1850 | 0.1388 | 0.4553 | 0.3415 | 0.4553 | 0.3415 | 0.4553 | 0.3415 | 0.4553 | 0.3415 |
| 37 | 0.4553 | 0.3415 | 0.2942 | 0.2206 | 0.1850 | 0.1388 | 0.4553 | 0.3415 | 0.4553 | 0.3415 | 0.4553 | 0.3415 | 0.4553 | 0.3415 |
| 38 | 0.4553 | 0.3415 | 0.2461 | 0.1845 | 0.1850 | 0.1388 | 0.4553 | 0.3415 | 0.4553 | 0.3415 | 0.4553 | 0.3415 | 0.4553 | 0.3415 |
| 39 | 0.4553 | 0.3415 | 0.1850 | 0.1388 | 0.1850 | 0.1388 | 0.4553 | 0.3415 | 0.4553 | 0.3415 | 0.4553 | 0.3415 | 0.4553 | 0.3415 |
| 40 | 1.0585 | 0.7939 | 0.2628 | 0.1971 | 0.2628 | 0.1971 | 0.6676 | 0.5007 | 1.0585 | 0.7939 | 1.0585 | 0.7939 | 1.0585 | 0.7939 |
| 41 | 1.0585 | 0.7939 | 0.2628 | 0.1971 | 0.2628 | 0.1971 | 0.6676 | 0.5007 | 1.0585 | 0.7939 | 1.0585 | 0.7939 | 1.0585 | 0.7939 |
| 42 | 1.0585 | 0.7939 | 0.2628 | 0.1971 | 0.2628 | 0.1971 | 0.6676 | 0.5007 | 1.0585 | 0.7939 | 1.0585 | 0.7939 | 1.0585 | 0.7939 |
| 43 | 1.0585 | 0.7939 | 0.2628 | 0.1971 | 0.2628 | 0.1971 | 0.4179 | 0.3134 | 1.0585 | 0.7939 | 1.0585 | 0.7939 | 1.0585 | 0.7939 |
| 44 | 1.0585 | 0.7939 | 0.2628 | 0.1971 | 0.2628 | 0.1971 | 0.4179 | 0.3134 | 1.0585 | 0.7939 | 1.0585 | 0.7939 | 1.0585 | 0.7939 |
| 45 | 1.8574 | 1.3931 | 0.5793 | 0.4344 | 0.5793 | 0.4344 | 0.9210 | 0.6908 | 1.8574 | 1.3931 | 1.8574 | 1.3931 | 1.8574 | 1.3931 |
| 46 | 1.8574 | 1.3931 | 0.5793 | 0.4344 | 0.5793 | 0.4344 | 0.9210 | 0.6908 | 1.8574 | 1.3931 | 1.8574 | 1.3931 | 1.8574 | 1.3931 |
| 47 | 1.8574 | 1.3931 | 0.5793 | 0.4344 | 0.5793 | 0.4344 | 0.9210 | 0.6908 | 1.8574 | 1.3931 | 1.8574 | 1.3931 | 1.8574 | 1.3931 |
| 48 | 1.8574 | 1.3931 | 0.5793 | 0.4344 | 0.5793 | 0.4344 | 0.9210 | 0.6908 | 1.8574 | 1.3931 | 1.8574 | 1.3931 | 1.8574 | 1.3931 |
| 49 | 1.8574 | 1.3931 | 0.5793 | 0.4344 | 0.5793 | 0.4344 | 0.9210 | 0.6908 | 1.8574 | 1.3931 | 1.8574 | 1.3931 | 1.8574 | 1.3931 |
| 50 | 2.6830 | 2.0122 | 0.9532 | 0.7149 | 0.9532 | 0.7149 | 1.2678 | 0.9509 | 2.4212 | 1.8159 | 2.6830 | 2.0122 | 2.6830 | 2.0122 |
| 51 | 2.6830 | 2.0122 | 0.9532 | 0.7149 | 0.9532 | 0.7149 | 1.2678 | 0.9509 | 2.4212 | 1.8159 | 2.6830 | 2.0122 | 2.6830 | 2.0122 |
| 52 | 2.6830 | 2.0122 | 0.9532 | 0.7149 | 0.9532 | 0.7149 | 1.2678 | 0.9509 | 2.4212 | 1.8159 | 2.6830 | 2.0122 | 2.6830 | 2.0122 |
| 53 | 2.6830 | 2.0122 | 0.9532 | 0.7149 | 0.9532 | 0.7149 | 1.2678 | 0.9509 | 2.4212 | 1.8159 | 2.6830 | 2.0122 | 2.6830 | 2.0122 |
| 54 | 2.6830 | 2.0122 | 0.9532 | 0.7149 | 0.9532 | 0.7149 | 1.2678 | 0.9509 | 2.4212 | 1.8159 | 2.6830 | 2.0122 | 2.6830 | 2.0122 |
| 55 | 4.3020 | 3.2265 | 1.2039 | 0.9029 | 1.2039 | 0.9029 | 1.3483 | 1.0112 | 2.4212 | 1.8159 | 4.3020 | 3.2265 | 4.3020 | 3.2265 |
| 56 | 4.3020 | 3.2265 | 1.2039 | 0.9029 | 1.2039 | 0.9029 | 1.3483 | 1.0112 | 2.4212 | 1.8159 | 4.3020 | 3.2265 | 4.3020 | 3.2265 |
| 57 | 4.3020 | 3.2265 | 1.2039 | 0.9029 | 1.2039 | 0.9029 | 1.3483 | 1.0112 | 2.4212 | 1.8159 | 4.3020 | 3.2265 | 4.3020 | 3.2265 |
| 58 | 4.3020 | 3.2265 | 1.2039 | 0.9029 | 1.2039 | 0.9029 | 1.3483 | 1.0112 | 2.4212 | 1.8159 | 4.3020 | 3.2265 | 4.3020 | 3.2265 |
| 59 | 4.3020 | 3.2265 | 1.2039 | 0.9029 | 1.2039 | 0.9029 | 1.3483 | 1.0112 | 2.4212 | 1.8159 | 4.3020 | 3.2265 | 4.3020 | 3.2265 |
| 60 | 5.5208 | 4.1406 | 2.0805 | 1.5604 | 2.0805 | 1.5604 | 2.0389 | 1.5291 | 2.7670 | 2.0753 | 5.2844 | 3.9633 | 5.5208 | 4.1406 |
| 61 | 5.5208 | 4.1406 | 2.0805 | 1.5604 | 2.0805 | 1.5604 | 2.0389 | 1.5291 | 2.7670 | 2.0753 | 5.2844 | 3.9633 | 5.5208 | 4.1406 |
| 62 | 5.5208 | 4.1406 | 2.0805 | 1.5604 | 2.0805 | 1.5604 | 2.0389 | 1.5291 | 2.7670 | 2.0753 | 5.2844 | 3.9633 | 5.5208 | 4.1406 |
| 63 | 5.5208 | 4.1406 | 2.0805 | 1.5604 | 2.0805 | 1.5604 | 2.0389 | 1.5291 | 2.7670 | 2.0753 | 5.2844 | 3.9633 | 5.5208 | 4.1406 |
| 64 | 5.5208 | 4.1406 | 2.0805 | 1.5604 | 2.0805 | 1.5604 | 2.0389 | 1.5291 | 2.7670 | 2.0753 | 5.2844 | 3.9633 | 5.5208 | 4.1406 |
| 65 | 7.2192 | 5.4144 | 4.0308 | 3.0231 | 4.0308 | 3.0231 | 4.0308 | 3.0231 | 4.5145 | 3.3858 | 6.4089 | 4.8067 | 7.2192 | 5.4144 |
| 66 | 7.2192 | 5.4144 | 4.0308 | 3.0231 | 4.0308 | 3.0231 | 4.0308 | 3.0231 | 4.5145 | 3.3858 | 6.4089 | 4.8067 | 7.2192 | 5.4144 |
| 67 | 7.2192 | 5.4144 | 4.0308 | 3.0231 | 4.0308 | 3.0231 | 4.0308 | 3.0231 | 4.5145 | 3.3858 | 6.4089 | 4.8067 | 7.2192 | 5.4144 |
| 68 | 7.2192 | 5.4144 | 4.0308 | 3.0231 | 4.0308 | 3.0231 | 4.0308 | 3.0231 | 4.5145 | 3.3858 | 6.4089 | 4.8067 | 7.2192 | 5.4144 |
| 69 | 7.2192 | 5.4144 | 4.0308 | 3.0231 | 4.0308 | 3.0231 | 4.0308 | 3.0231 | 4.5145 | 3.3858 | 6.4089 | 4.8067 | 7.2192 | 5.4144 |
| 70 | 11.5953 | 8.6964 | 6.6620 | 4.9965 | 6.6620 | 4.9965 | 6.6620 | 4.9965 | 6.6620 | 4.9965 | 8.8605 | 6.6454 | 11.5953 | 8.6964 |
| 71 | 11.5953 | 8.6964 | 6.6620 | 4.9965 | 6.6620 | 4.9965 | 6.6620 | 4.9965 | 6.6620 | 4.9965 | 8.8605 | 6.6454 | 11.5953 | 8.6964 |
| 72 | 11.5953 | 8.6964 | 6.6620 | 4.9965 | 6.6620 | 4.9965 | 6.6620 | 4.9965 | 6.6620 | 4.9965 | 8.8605 | 6.6454 | 11.5953 | 8.6964 |
| 73 | 11.5953 | 8.6964 | 6.6620 | 4.9965 | 6.6620 | 4.9965 | 6.6620 | 4.9965 | 6.6620 | 4.9965 | 8.8605 | 6.6454 | 11.5953 | 8.6964 |
| 74 | 11.5953 | 8.6964 | 6.6620 | 4.9965 | 6.6620 | 4.9965 | 6.6620 | 4.9965 | 6.6620 | 4.9965 | 8.8605 | 6.6454 | 11.5953 | 8.6964 |
| 75 | 22.8185 | 17.1139 | 15.3559 | 11.5169 | 15.3559 | 11.5169 | 15.3559 | 11.5169 | 15.3559 | 11.5169 | 17.1986 | 12.8989 | 22.8185 | 17.1139 |
| 76 | 22.8185 | 17.1139 | 15.3559 | 11.5169 | 15.3559 | 11.5169 | 15.3559 | 11.5169 | 15.3559 | 11.5169 | 17.1986 | 12.8989 | 22.8185 | 17.1139 |
| 77 | 22.8185 | 17.1139 | 15.3559 | 11.5169 | 15.3559 | 11.5169 | 15.3559 | 11.5169 | 15.3559 | 11.5169 | 17.1986 | 12.8989 | 22.8185 | 17.1139 |
| 78 | 22.8185 | 17.1139 | 15.3559 | 11.5169 | 15.3559 | 11.5169 | 15.3559 | 11.5169 | 15.3559 | 11.5169 | 15.0487 | 11.2866 | 22.8185 | 17.1139 |
| 79 | 22.8185 | 17.1139 | 15.3559 | 11.5169 | 15.3559 | 11.5169 | 15.3559 | 11.5169 | 15.3559 | 11.5169 | 15.0487 | 11.2866 | 22.8185 | 17.1139 |
| 80 | 51.0234 | 38.2675 | 33.1797 | 24.8848 | 33.1797 | 24.8848 | 33.1797 | 24.8848 | 33.1797 | 24.8848 | 33.1797 | 24.8848 | 44.1290 | 33.0968 |
| 81 | 51.0234 | 38.2675 | 33.1797 | 24.8848 | 33.1797 | 24.8848 | 33.1797 | 24.8848 | 33.1797 | 24.8848 | 33.1797 | 24.8848 | 44.1290 | 33.0968 |
| 82 | 51.0234 | 38.2675 | 33.1797 | 24.8848 | 33.1797 | 24.8848 | 33.1797 | 24.8848 | 33.1797 | 24.8848 | 33.1797 | 24.8848 | 44.1290 | 33.0968 |
| 83 | 51.0234 | 38.2675 | 33.1797 | 24.8848 | 33.1797 | 24.8848 | 33.1797 | 24.8848 | 33.1797 | 24.8848 | 33.1797 | 24.8848 | 44.1290 | 33.0968 |
| 84 | 51.0234 | 38.2675 | 33.1797 | 24.8848 | 33.1797 | 24.8848 | 33.1797 | 24.8848 | 33.1797 | 24.8848 | 33.1797 | 24.8848 | 44.1290 | 33.0968 |
| 85 | 65.8187 | 57.5914 | 33.1797 | 29.0323 | 33.1797 | 29.0323 | 33.1797 | 29.0323 | 44.3800 | 38.8325 | 44.3800 | 38.8325 | 44.3800 | 38.8325 |
| 86 | 65.8187 | 57.5914 | 33.1797 | 29.0323 | 33.1797 | 29.0323 | 33.1797 | 29.0323 | 44.3800 | 38.8325 | 44.3800 | 38.8325 | 44.3800 | 38.8325 |
| 87 | 65.8187 | 57.5914 | 33.1797 | 29.0323 | 33.1797 | 29.0323 | 33.1797 | 29.0323 | 44.3800 | 38.8325 | 44.3800 | 38.8325 | 44.3800 | 38.8325 |
| 88 | 65.8187 | 57.5914 | 33.1797 | 29.0323 | 33.1797 | 29.0323 | 33.1797 | 29.0323 | 44.3800 | 38.8325 | 44.3800 | 38.8325 | 44.3800 | 38.8325 |
| 89 | 65.8187 | 57.5914 | 33.1797 | 29.0323 | 33.1797 | 29.0323 | 33.1797 | 29.0323 | 44.3800 | 38.8325 | 44.3800 | 38.8325 | 44.3800 | 38.8325 |
| 90 | 65.8187 | 57.5914 | 33.1797 | 29.0323 | 33.1797 | 29.0323 | 44.3800 | 38.8325 | 44.3800 | 38.8325 | 44.3800 | 38.8325 | 44.3800 | 38.8325 |
| 91 | 65.8187 | 57.5914 | 33.1797 | 29.0323 | 33.1797 | 29.0323 | 44.3800 | 38.8325 | 44.3800 | 38.8325 | 44.3800 | 38.8325 | 44.3800 | 38.8325 |
| 92 | 65.8187 | 57.5914 | 33.1797 | 29.0323 | 33.1797 | 29.0323 | 44.3800 | 38.8325 | 44.3800 | 38.8325 | 44.3800 | 38.8325 | 44.3800 | 38.8325 |
| 93 | 65.8187 | 57.5914 | 33.1797 | 29.0323 | 33.1797 | 29.0323 | 44.3800 | 38.8325 | 44.3800 | 38.8325 | 44.3800 | 38.8325 | 44.3800 | 38.8325 |
| 94 | 65.8187 | 57.5914 | 33.1797 | 29.0323 | 33.1797 | 29.0323 | 44.3800 | 38.8325 | 44.3800 | 38.8325 | 44.3800 | 38.8325 | 44.3800 | 38.8325 |
| 95 | 65.8187 | 57.5914 | 33.1797 | 29.0323 | 33.1797 | 29.0323 | 44.3800 | 38.8325 | 44.3800 | 38.8325 | 44.3800 | 38.8325 | 44.3800 | 38.8325 |
| 96 | 65.8187 | 57.5914 | 33.1797 | 29.0323 | 33.1797 | 29.0323 | 44.3800 | 38.8325 | 44.3800 | 38.8325 | 44.3800 | 38.8325 | 44.3800 | 38.8325 |
| 97 | 65.8187 | 57.5914 | 33.1797 | 29.0323 | 33.1797 | 29.0323 | 44.3800 | 38.8325 | 44.3800 | 38.8325 | 44.3800 | 38.8325 | 44.3800 | 38.8325 |
| 98 | 65.8187 | 57.5914 | 33.1797 | 29.0323 | 33.1797 | 29.0323 | 44.3800 | 38.8325 | 44.3800 | 38.8325 | 44.3800 | 38.8325 | 44.3800 | 38.8325 |
| 99 | 65.8187 | 57.5914 | 33.1797 | 29.0323 | 33.1797 | 29.0323 | 44.3800 | 38.8325 | 44.3800 | 38.8325 | 44.3800 | 38.8325 | 44.3800 | 38.8325 |
| 100 | 65.8187 | 57.5914 | 33.1797 | 29.0323 | 33.1797 | 29.0323 | 44.3800 | 38.8325 | 44.3800 | 38.8325 | 44.3800 | 38.8325 | 44.3800 | 38.8325 |

References:

1. Doll R, Peto R, Wheatley K, Gray R, Sutherland I (1994) Mortality in relation to smoking: 40 years’ observations on male British doctors. BMJ 309: 901–911. doi:10.1136/bmj.309.6959.901.

2. Rasmussen SR, Prescott E, Sørensen TI, Søgaard J (2004) The total lifetime costs of smoking. The European Journal of Public Health 14: 95–100.

3. WHO (n.d.) REPORT On THE global tobacoo epidemic, 2011. WHO. Available: http://whqlibdoc.who.int/publications/2011/9789240687813_eng.pdf?ua=1.

4. Peto R, Lopez AD, Boreham J, Thun M (2006) Mortality from smoking in developed countries 1950-2000 (2nd edition). Available: http://www.ctsu.ox.ac.uk/deathsfromsmoking/download%20files/Original%20research/Mortality%20from%20smoking%20in%20developed%20countries%201950-2000%20%282nd%20ed.%29.pdf.
