# Supplementary material for: Cost Effectiveness of Free Access to Smoking Cessation Treatment in France Considering the Economic Burden of Smoking-Related Diseases
Source: PLoS One. 2016 Feb 24;11(2):e0148750. doi: 10.1371/journal.pone.0148750 (PMC4766094; doi:10.1371/journal.pone.0148750)
Supplement: S4 Table — (DOCX) [file pone.0148750.s004.docx]

S4 Table: Overview of adjusted mortality rate from others causes used in the model

| **Annual mortality rate(per 1000) for others causes stratified by age, gender and time since cessation** | | | | | | | | | | | | | | |
| --- | --- | --- | --- | --- | --- | --- | --- | --- | --- | --- | --- | --- | --- | --- |
| **Age** | **Smoker** | | **Former aged 15-24** | | **Former aged 25-34** | | **Former aged 35-44** | | **Former aged 45-54** | | **Former aged 55-64** | | **Former aged 65-74** | |
|  | M | F | M | F | M | F | M | F | M | F | M | F | M | F |
| 15 | 0.4800 | 0.2000 | 0.4800 | 0.2000 | 0.4800 | 0.2000 | 0.4800 | 0.2000 | 0.4800 | 0.2000 | 0.4800 | 0.2000 | 0.4800 | 0.2000 |
| 16 | 0.4800 | 0.2000 | 0.4800 | 0.2000 | 0.4800 | 0.2000 | 0.4800 | 0.2000 | 0.4800 | 0.2000 | 0.4800 | 0.2000 | 0.4800 | 0.2000 |
| 17 | 0.4800 | 0.2000 | 0.4800 | 0.2000 | 0.4800 | 0.2000 | 0.4800 | 0.2000 | 0.4800 | 0.2000 | 0.4800 | 0.2000 | 0.4800 | 0.2000 |
| 18 | 0.4800 | 0.2000 | 0.4800 | 0.2000 | 0.4800 | 0.2000 | 0.4800 | 0.2000 | 0.4800 | 0.2000 | 0.4800 | 0.2000 | 0.4800 | 0.2000 |
| 19 | 0.4800 | 0.2000 | 0.4800 | 0.2000 | 0.4800 | 0.2000 | 0.4800 | 0.2000 | 0.4800 | 0.2000 | 0.4800 | 0.2000 | 0.4800 | 0.2000 |
| 20 | 0.8900 | 0.2900 | 0.8900 | 0.2900 | 0.8900 | 0.2900 | 0.8900 | 0.2900 | 0.8900 | 0.2900 | 0.8900 | 0.2900 | 0.8900 | 0.2900 |
| 21 | 0.8900 | 0.2900 | 0.8900 | 0.2900 | 0.8900 | 0.2900 | 0.8900 | 0.2900 | 0.8900 | 0.2900 | 0.8900 | 0.2900 | 0.8900 | 0.2900 |
| 22 | 0.8900 | 0.2900 | 0.8900 | 0.2900 | 0.8900 | 0.2900 | 0.8900 | 0.2900 | 0.8900 | 0.2900 | 0.8900 | 0.2900 | 0.8900 | 0.2900 |
| 23 | 0.8900 | 0.2900 | 0.8900 | 0.2900 | 0.8900 | 0.2900 | 0.8900 | 0.2900 | 0.8900 | 0.2900 | 0.8900 | 0.2900 | 0.8900 | 0.2900 |
| 24 | 0.8900 | 0.2900 | 0.8900 | 0.2900 | 0.8900 | 0.2900 | 0.8900 | 0.2900 | 0.8900 | 0.2900 | 0.8900 | 0.2900 | 0.8900 | 0.2900 |
| 25 | 0.9100 | 0.3000 | 0.9100 | 0.3000 | 0.9100 | 0.3000 | 0.9100 | 0.3000 | 0.9100 | 0.3000 | 0.9100 | 0.3000 | 0.9100 | 0.3000 |
| 26 | 0.9100 | 0.3000 | 0.9100 | 0.3000 | 0.9100 | 0.3000 | 0.9100 | 0.3000 | 0.9100 | 0.3000 | 0.9100 | 0.3000 | 0.9100 | 0.3000 |
| 27 | 0.9100 | 0.3000 | 0.9100 | 0.3000 | 0.9100 | 0.3000 | 0.9100 | 0.3000 | 0.9100 | 0.3000 | 0.9100 | 0.3000 | 0.9100 | 0.3000 |
| 28 | 0.9100 | 0.3000 | 0.9100 | 0.3000 | 0.9100 | 0.3000 | 0.9100 | 0.3000 | 0.9100 | 0.3000 | 0.9100 | 0.3000 | 0.9100 | 0.3000 |
| 29 | 0.9100 | 0.3000 | 0.9100 | 0.3000 | 0.9100 | 0.3000 | 0.9100 | 0.3000 | 0.9100 | 0.3000 | 0.9100 | 0.3000 | 0.9100 | 0.3000 |
| 30 | 1.0900 | 0.4500 | 1.0900 | 0.4500 | 1.0900 | 0.4500 | 1.0900 | 0.4500 | 1.0900 | 0.4500 | 1.0900 | 0.4500 | 1.0900 | 0.4500 |
| 31 | 1.0900 | 0.4500 | 1.0900 | 0.4500 | 1.0900 | 0.4500 | 1.0900 | 0.4500 | 1.0900 | 0.4500 | 1.0900 | 0.4500 | 1.0900 | 0.4500 |
| 32 | 1.0900 | 0.4500 | 1.0900 | 0.4500 | 1.0900 | 0.4500 | 1.0900 | 0.4500 | 1.0900 | 0.4500 | 1.0900 | 0.4500 | 1.0900 | 0.4500 |
| 33 | 1.0900 | 0.4500 | 1.0900 | 0.4500 | 1.0900 | 0.4500 | 1.0900 | 0.4500 | 1.0900 | 0.4500 | 1.0900 | 0.4500 | 1.0900 | 0.4500 |
| 34 | 1.0900 | 0.4500 | 1.0900 | 0.4500 | 1.0900 | 0.4500 | 1.0900 | 0.4500 | 1.0900 | 0.4500 | 1.0900 | 0.4500 | 1.0900 | 0.4500 |
| 35 | 2.1056 | 1.5792 | 1.5954 | 1.1965 | 2.2667 | 1.7001 | 2.1056 | 1.5792 | 2.1056 | 1.5792 | 2.1056 | 1.5792 | 2.1056 | 1.5792 |
| 36 | 2.1056 | 1.5792 | 1.5954 | 1.1965 | 2.2667 | 1.7001 | 2.1056 | 1.5792 | 2.1056 | 1.5792 | 2.1056 | 1.5792 | 2.1056 | 1.5792 |
| 37 | 2.1056 | 1.5792 | 1.5954 | 1.1965 | 2.2667 | 1.7001 | 2.1056 | 1.5792 | 2.1056 | 1.5792 | 2.1056 | 1.5792 | 2.1056 | 1.5792 |
| 38 | 2.1056 | 1.5792 | 1.5954 | 1.1965 | 2.1997 | 1.6498 | 2.1056 | 1.5792 | 2.1056 | 1.5792 | 2.1056 | 1.5792 | 2.1056 | 1.5792 |
| 39 | 2.1056 | 1.5792 | 1.5954 | 1.1965 | 2.3057 | 1.7293 | 2.1056 | 1.5792 | 2.1056 | 1.5792 | 2.1056 | 1.5792 | 2.1056 | 1.5792 |
| 40 | 3.4847 | 2.6135 | 2.1996 | 1.6497 | 2.1996 | 1.6497 | 3.8795 | 2.9096 | 3.4847 | 2.6135 | 3.4847 | 2.6135 | 3.4847 | 2.6135 |
| 41 | 3.4847 | 2.6135 | 2.1996 | 1.6497 | 2.1996 | 1.6497 | 3.8795 | 2.9096 | 3.4847 | 2.6135 | 3.4847 | 2.6135 | 3.4847 | 2.6135 |
| 42 | 3.4847 | 2.6135 | 2.1996 | 1.6497 | 2.1996 | 1.6497 | 3.8795 | 2.9096 | 3.4847 | 2.6135 | 3.4847 | 2.6135 | 3.4847 | 2.6135 |
| 43 | 3.4847 | 2.6135 | 2.1996 | 1.6497 | 2.1996 | 1.6497 | 3.4980 | 2.6235 | 3.4847 | 2.6135 | 3.4847 | 2.6135 | 3.4847 | 2.6135 |
| 44 | 3.4847 | 2.6135 | 2.1996 | 1.6497 | 2.1996 | 1.6497 | 3.4980 | 2.6235 | 3.4847 | 2.6135 | 3.4847 | 2.6135 | 3.4847 | 2.6135 |
| 45 | 5.3271 | 3.9954 | 2.1619 | 1.6214 | 2.1619 | 1.6214 | 3.3232 | 2.4924 | 5.3271 | 3.9954 | 5.3271 | 3.9954 | 5.3271 | 3.9954 |
| 46 | 5.3271 | 3.9954 | 2.1619 | 1.6214 | 2.1619 | 1.6214 | 3.3232 | 2.4924 | 5.3271 | 3.9954 | 5.3271 | 3.9954 | 5.3271 | 3.9954 |
| 47 | 5.3271 | 3.9954 | 2.1619 | 1.6214 | 2.1619 | 1.6214 | 3.3232 | 2.4924 | 5.3271 | 3.9954 | 5.3271 | 3.9954 | 5.3271 | 3.9954 |
| 48 | 5.3271 | 3.9954 | 2.1619 | 1.6214 | 2.1619 | 1.6214 | 3.3232 | 2.4924 | 5.3271 | 3.9954 | 5.3271 | 3.9954 | 5.3271 | 3.9954 |
| 49 | 5.3271 | 3.9954 | 2.1619 | 1.6214 | 2.1619 | 1.6214 | 3.3232 | 2.4924 | 5.3271 | 3.9954 | 5.3271 | 3.9954 | 5.3271 | 3.9954 |
| 50 | 10.3402 | 7.7552 | 3.8255 | 2.8691 | 3.8255 | 2.8691 | 4.8826 | 3.6619 | 7.4637 | 5.5978 | 10.3402 | 7.7552 | 10.3402 | 7.7552 |
| 51 | 10.3402 | 7.7552 | 3.8255 | 2.8691 | 3.8255 | 2.8691 | 4.8826 | 3.6619 | 7.4637 | 5.5978 | 10.3402 | 7.7552 | 10.3402 | 7.7552 |
| 52 | 10.3402 | 7.7552 | 3.8255 | 2.8691 | 3.8255 | 2.8691 | 4.8826 | 3.6619 | 7.4637 | 5.5978 | 10.3402 | 7.7552 | 10.3402 | 7.7552 |
| 53 | 10.3402 | 7.7552 | 3.8255 | 2.8691 | 3.8255 | 2.8691 | 4.8826 | 3.6619 | 7.4637 | 5.5978 | 10.3402 | 7.7552 | 10.3402 | 7.7552 |
| 54 | 10.3402 | 7.7552 | 3.8255 | 2.8691 | 3.8255 | 2.8691 | 4.8826 | 3.6619 | 7.4637 | 5.5978 | 10.3402 | 7.7552 | 10.3402 | 7.7552 |
| 55 | 10.0758 | 7.5568 | 4.4104 | 3.3078 | 4.4104 | 3.3078 | 4.5963 | 3.4472 | 7.4157 | 5.5618 | 10.0758 | 7.5568 | 10.0758 | 7.5568 |
| 56 | 10.0758 | 7.5568 | 4.4104 | 3.3078 | 4.4104 | 3.3078 | 4.5963 | 3.4472 | 7.4157 | 5.5618 | 10.0758 | 7.5568 | 10.0758 | 7.5568 |
| 57 | 10.0758 | 7.5568 | 4.4104 | 3.3078 | 4.4104 | 3.3078 | 4.5963 | 3.4472 | 7.4157 | 5.5618 | 10.0758 | 7.5568 | 10.0758 | 7.5568 |
| 58 | 10.0758 | 7.5568 | 4.4104 | 3.3078 | 4.4104 | 3.3078 | 4.5963 | 3.4472 | 7.4157 | 5.5618 | 10.0758 | 7.5568 | 10.0758 | 7.5568 |
| 59 | 10.0758 | 7.5568 | 4.4104 | 3.3078 | 4.4104 | 3.3078 | 4.5963 | 3.4472 | 7.4157 | 5.5618 | 10.0758 | 7.5568 | 10.0758 | 7.5568 |
| 60 | 13.2220 | 9.9165 | 5.8850 | 4.4137 | 5.8850 | 4.4137 | 5.4025 | 4.0519 | 7.0699 | 5.3024 | 12.6836 | 9.5127 | 13.2220 | 9.9165 |
| 61 | 13.2220 | 9.9165 | 5.8850 | 4.4137 | 5.8850 | 4.4137 | 5.4025 | 4.0519 | 7.0699 | 5.3024 | 12.6836 | 9.5127 | 13.2220 | 9.9165 |
| 62 | 13.2220 | 9.9165 | 5.8850 | 4.4137 | 5.8850 | 4.4137 | 5.4025 | 4.0519 | 7.0699 | 5.3024 | 12.6836 | 9.5127 | 13.2220 | 9.9165 |
| 63 | 13.2220 | 9.9165 | 5.8850 | 4.4137 | 5.8850 | 4.4137 | 5.4025 | 4.0519 | 7.0699 | 5.3024 | 5.5214 | 4.1410 | 13.2220 | 9.9165 |
| 64 | 13.2220 | 9.9165 | 5.8850 | 4.4137 | 5.8850 | 4.4137 | 5.4025 | 4.0519 | 7.0699 | 5.3024 | 5.5214 | 4.1410 | 13.2220 | 9.9165 |
| 65 | 16.2539 | 12.1904 | 7.6556 | 5.7417 | 7.6556 | 5.7417 | 7.4184 | 5.5638 | 7.9242 | 5.9431 | 9.9653 | 7.4740 | 16.2539 | 12.1904 |
| 66 | 16.2539 | 12.1904 | 7.6556 | 5.7417 | 7.6556 | 5.7417 | 7.4184 | 5.5638 | 7.9242 | 5.9431 | 9.9653 | 7.4740 | 16.2539 | 12.1904 |
| 67 | 16.2539 | 12.1904 | 7.6556 | 5.7417 | 7.6556 | 5.7417 | 7.4184 | 5.5638 | 7.9242 | 5.9431 | 9.9653 | 7.4740 | 16.2539 | 12.1904 |
| 68 | 16.2539 | 12.1904 | 7.6556 | 5.7417 | 7.6556 | 5.7417 | 7.1498 | 5.3623 | 6.1067 | 4.5800 | 9.9653 | 7.4740 | 16.2539 | 12.1904 |
| 69 | 16.2539 | 12.1904 | 7.6556 | 5.7417 | 7.6556 | 5.7417 | 7.1498 | 5.3623 | 6.1067 | 4.5800 | 9.9653 | 7.4740 | 16.2539 | 12.1904 |
| 70 | 21.4849 | 16.1137 | 11.7949 | 8.8462 | 11.7949 | 8.8462 | 11.3041 | 8.4781 | 10.7447 | 8.0585 | 13.3896 | 10.0422 | 22.9516 | 17.2137 |
| 71 | 21.4849 | 16.1137 | 11.7949 | 8.8462 | 11.7949 | 8.8462 | 11.3041 | 8.4781 | 10.7447 | 8.0585 | 13.3896 | 10.0422 | 22.9516 | 17.2137 |
| 72 | 21.4849 | 16.1137 | 11.7949 | 8.8462 | 11.7949 | 8.8462 | 11.3041 | 8.4781 | 10.7447 | 8.0585 | 13.3896 | 10.0422 | 22.9516 | 17.2137 |
| 73 | 21.4849 | 16.1137 | 11.7949 | 8.8462 | 11.7949 | 8.8462 | 10.7502 | 8.0627 | 10.6248 | 7.9686 | 8.8531 | 6.6398 | 24.8859 | 18.6645 |
| 74 | 21.4849 | 16.1137 | 11.7949 | 8.8462 | 11.7949 | 8.8462 | 10.7502 | 8.0627 | 10.6248 | 7.9686 | 8.8531 | 6.6398 | 24.8859 | 18.6645 |
| 75 | 33.9605 | 25.4704 | 19.5569 | 14.6677 | 19.5569 | 14.6677 | 18.4383 | 13.8287 | 18.5008 | 13.8756 | 18.7383 | 14.0537 | 30.8828 | 23.1621 |
| 76 | 33.9605 | 25.4704 | 19.5569 | 14.6677 | 19.5569 | 14.6677 | 18.4383 | 13.8287 | 18.5008 | 13.8756 | 18.7383 | 14.0537 | 30.8828 | 23.1621 |
| 77 | 33.9605 | 25.4704 | 19.5569 | 14.6677 | 19.5569 | 14.6677 | 18.4383 | 13.8287 | 18.5008 | 13.8756 | 18.7383 | 14.0537 | 30.8828 | 23.1621 |
| 78 | 33.9605 | 25.4704 | 19.5569 | 14.6677 | 19.5569 | 14.6677 | 18.4383 | 13.8287 | 18.4689 | 13.8517 | 20.8881 | 15.6661 | 32.4659 | 24.3494 |
| 79 | 33.9605 | 25.4704 | 19.5569 | 14.6677 | 19.5569 | 14.6677 | 18.4383 | 13.8287 | 18.4689 | 13.8517 | 20.8881 | 15.6661 | 32.4659 | 24.3494 |
| 80 | 48.7266 | 36.5449 | 34.0721 | 25.5541 | 34.0721 | 25.5541 | 32.9188 | 24.6891 | 32.9188 | 24.6891 | 27.9951 | 20.9963 | 43.5903 | 32.6927 |
| 81 | 48.7266 | 36.5449 | 34.0721 | 25.5541 | 34.0721 | 25.5541 | 32.9188 | 24.6891 | 32.9188 | 24.6891 | 27.9951 | 20.9963 | 43.5903 | 32.6927 |
| 82 | 48.7266 | 36.5449 | 34.0721 | 25.5541 | 34.0721 | 25.5541 | 32.9188 | 24.6891 | 32.9188 | 24.6891 | 27.9951 | 20.9963 | 43.5903 | 32.6927 |
| 83 | 48.7266 | 36.5449 | 34.0721 | 25.5541 | 34.0721 | 25.5541 | 32.9188 | 24.6891 | 32.4842 | 24.3632 | 27.9951 | 20.9963 | 43.5903 | 32.6927 |
| 84 | 48.7266 | 36.5449 | 34.0721 | 25.5541 | 34.0721 | 25.5541 | 32.9188 | 24.6891 | 32.4842 | 24.3632 | 27.9951 | 20.9963 | 43.5903 | 32.6927 |
| 85 | 33.0942 | 24.8206 | 91.2843 | 68.4632 | 91.2843 | 68.4632 | 90.1414 | 67.6061 | 74.0000 | 55.5000 | 74.0000 | 55.5000 | 62.2599 | 46.6950 |
| 86 | 33.0942 | 24.8206 | 91.2843 | 68.4632 | 91.2843 | 68.4632 | 90.1414 | 67.6061 | 74.0000 | 55.5000 | 74.0000 | 55.5000 | 62.2599 | 46.6950 |
| 87 | 33.0942 | 24.8206 | 91.2843 | 68.4632 | 91.2843 | 68.4632 | 90.1414 | 67.6061 | 74.0000 | 55.5000 | 74.0000 | 55.5000 | 62.2599 | 46.6950 |
| 88 | 33.0942 | 24.8206 | 91.2843 | 68.4632 | 91.2843 | 68.4632 | 90.1414 | 67.6061 | 74.0000 | 55.5000 | 74.0000 | 55.5000 | 62.2599 | 46.6950 |
| 89 | 33.0942 | 24.8206 | 91.2843 | 68.4632 | 91.2843 | 68.4632 | 90.1414 | 67.6061 | 74.0000 | 55.5000 | 74.0000 | 55.5000 | 62.2599 | 46.6950 |
| 90 | 90.7405 | 68.0554 | 91.2843 | 68.4632 | 91.2843 | 68.4632 | 78.9411 | 59.2058 | 74.0000 | 55.5000 | 74.0000 | 55.5000 | 71.4946 | 53.6210 |
| 91 | 90.7405 | 68.0554 | 91.2843 | 68.4632 | 91.2843 | 68.4632 | 78.9411 | 59.2058 | 74.0000 | 55.5000 | 74.0000 | 55.5000 | 71.4946 | 53.6210 |
| 92 | 90.7405 | 68.0554 | 91.2843 | 68.4632 | 91.2843 | 68.4632 | 78.9411 | 59.2058 | 74.0000 | 55.5000 | 74.0000 | 55.5000 | 71.4946 | 53.6210 |
| 93 | 90.7405 | 68.0554 | 91.2843 | 68.4632 | 91.2843 | 68.4632 | 78.9411 | 59.2058 | 74.0000 | 55.5000 | 74.0000 | 55.5000 | 68.9893 | 51.7419 |
| 94 | 90.7405 | 68.0554 | 91.2843 | 68.4632 | 91.2843 | 68.4632 | 78.9411 | 59.2058 | 74.0000 | 55.5000 | 74.0000 | 55.5000 | 68.9893 | 51.7419 |
| 95 | 90.7405 | 68.0554 | 91.2843 | 68.4632 | 91.2843 | 68.4632 | 78.9411 | 59.2058 | 74.0000 | 55.5000 | 74.0000 | 55.5000 | 68.9893 | 51.7419 |
| 96 | 90.7405 | 68.0554 | 91.2843 | 68.4632 | 91.2843 | 68.4632 | 78.9411 | 59.2058 | 74.0000 | 55.5000 | 74.0000 | 55.5000 | 68.9893 | 51.7419 |
| 97 | 90.7405 | 68.0554 | 91.2843 | 68.4632 | 91.2843 | 68.4632 | 78.9411 | 59.2058 | 74.0000 | 55.5000 | 74.0000 | 55.5000 | 68.9893 | 51.7419 |
| 98 | 90.7405 | 68.0554 | 91.2843 | 68.4632 | 91.2843 | 68.4632 | 78.9411 | 59.2058 | 74.0000 | 55.5000 | 74.0000 | 55.5000 | 68.9893 | 51.7419 |
| 99 | 90.7405 | 68.0554 | 91.2843 | 68.4632 | 91.2843 | 68.4632 | 78.9411 | 59.2058 | 74.0000 | 55.5000 | 74.0000 | 55.5000 | 68.9893 | 51.7419 |
| 100 | 91.9566 | 68.9675 | 139.4263 | 104.5697 | 139.4263 | 104.5697 | 127.0832 | 95.3124 | 122.1420 | 91.6065 | 122.1420 | 91.6065 | 106.5351 | 79.9013 |

References:

1. Doll R, Peto R, Wheatley K, Gray R, Sutherland I (1994) Mortality in relation to smoking: 40 years’ observations on male British doctors. BMJ 309: 901–911. doi:10.1136/bmj.309.6959.901.

2. Rasmussen SR, Prescott E, Sørensen TI, Søgaard J (2004) The total lifetime costs of smoking. The European Journal of Public Health 14: 95–100.

3. WHO (n.d.) REPORT On THE global tobacoo epidemic, 2011. WHO. Available: http://whqlibdoc.who.int/publications/2011/9789240687813_eng.pdf?ua=1.

4. Peto R, Lopez AD, Boreham J, Thun M (2006) Mortality from smoking in developed countries 1950-2000 (2nd edition). Available: http://www.ctsu.ox.ac.uk/deathsfromsmoking/download%20files/Original%20research/Mortality%20from%20smoking%20in%20developed%20countries%201950-2000%20%282nd%20ed.%29.pdf.
